# Modulation of oxidative phosphorylation augments antineoplastic activity of mitotic aurora kinase inhibition

**DOI:** 10.1038/s41419-021-04190-w

**Published:** 2021-09-30

**Authors:** Zijian Zhang, Deshun Zeng, Wei Zhang, Ailin Chen, Jie Lei, Fang Liu, Bing Deng, Junxiao Zhuo, Bin He, Min Yan, Xinxing Lei, Shulan Wang, Eric W.-F. Lam, Quentin Liu, Zifeng Wang

**Affiliations:** 1 Sun Yat-sen University Cancer Center; State Key Laboratory of Oncology in South China; Collaborative Innovation Center for Cancer Medicine, Guangzhou, 510060 China; 2 Department of Clinical Immunology, The Third Affiliated Hospital, Sun Yat-sen University, Guangzhou, 510630 China; 3 Seventh Affiliated Hospital, Sun Yat-sen University, Shenzhen, 518107 China; 4 Institute of Cancer Stem Cell, Dalian Medical University, Dalian, 116044 China

**Keywords:** Mitosis, Cancer screening, Preclinical research

## Abstract

Uncontrolled mitosis is one of the most important features of cancer, and mitotic kinases are thought to be ideal targets for anticancer therapeutics. However, despite numerous clinical attempts spanning decades, clinical trials for mitotic kinase-targeting agents have generally stalled in the late stages due to limited therapeutic effectiveness. Alisertib (MLN8237) is a promising oral mitotic aurora kinase A (*AURKA*, Aurora-A) selective inhibitor, which is currently under several clinical evaluations but has failed in its first Phase III trial due to inadequate efficacy. In this study, we performed genome-wide CRISPR/Cas9-based screening to identify vulnerable biological processes associated with alisertib in breast cancer MDA-MB-231 cells. The result indicated that alisertib treated cancer cells are more sensitive to the genetic perturbation of oxidative phosphorylation (OXPHOS). Mechanistic investigation indicated that alisertib treatment, as well as other mitotic kinase inhibitors, rapidly reduces the intracellular ATP level to generate a status that is highly addictive to OXPHOS. Furthermore, the combinational inhibition of mitotic kinase and OXPHOS by alisertib, and metformin respectively, generates severe energy exhaustion in mitotic cells that consequently triggers cell death. The combination regimen also enhanced tumor regression significantly in vivo. This suggests that targeting OXPHOS by metformin is a potential strategy for promoting the therapeutic effects of mitotic kinase inhibitors through the joint targeting of mitosis and cellular energy homeostasis.

## Introduction

Mitosis is a key step in the cell cycle, and it governs the production of new daughter cells from old ones. During this process, mitotic kinases, such as CDK1, Aurora-A, Aurora-B, and PLK1, are central players that drive entry into and progression through the division phase [1, 2]. The dysregulation of mitotic kinases is widely associated with uncontrolled cell proliferation and cancer development [1, 3, 4]. Given the critical role in cell proliferation and cancer progression, mitotic kinases are believed to be ideal targets for cancer therapy. Numerous mitotic inhibitors have been developed and evaluated in a number of preclinical and clinical studies [3–5].

Alisertib is a representative mitotic kinase inhibitor that is currently under investigation in a number of hematological and solid cancers [6]. It is a selective orally administered small molecule inhibitor targeting Aurora-A. Aurora-A overexpression and/or amplification have been detected in multiple cancer types and are widely associated with drug resistance, tumor progression, and poor prognosis [7–9]. Alisertib shares the advantages of other mitotic kinase inhibitors over cytotoxic chemotherapy predecessors as it has minimal toxicity in non-proliferating tissues. However, the first randomized Phase III trial of alisertib (NCT01482962) was halted as it failed to demonstrate superiority in patients with relapsed or refractory peripheral T-cell lymphoma [10]. These findings suggest that further investigations are required to improve the antineoplastic effect for the development of alisertib as a clinically efficacious anticancer agent.

Combination therapy is a way to increase treatment efficacy and tolerance. To explore a potential clinically applicable combination regimen, we performed genome-wide CRISPR knockout screening and found that alisertib treated cells are more sensitive to oxidative phosphorylation (OXPHOS) inhibition. Alisertib, as well as CDK1 and PLK1 inhibitors, reduced cellular energy levels during mitosis. We further interrogated the combination effects of OXPHOS inhibitors, such as an inhibitor of complex I of the electron transport chain (ETC) metformin and mitotic kinase inhibitor alisertib, and discovered that the enhanced anticancer effect is a result of an increased mitotic failure caused by energy homeostasis disruption. The combination of metformin and alisertib in vivo has shown strong tumor suppressive effects accompanied by minor body weight loss. This suggests that a combination of OXPHOS inhibitors such as metformin with mitotic inhibitors may be a novel and effective option for cancer therapy.

## Results

### CRISPR/Cas9-based genome-wide screening reveals cellular OXPHOS-dependence upon alisertib treatment

**Fig. 1:**
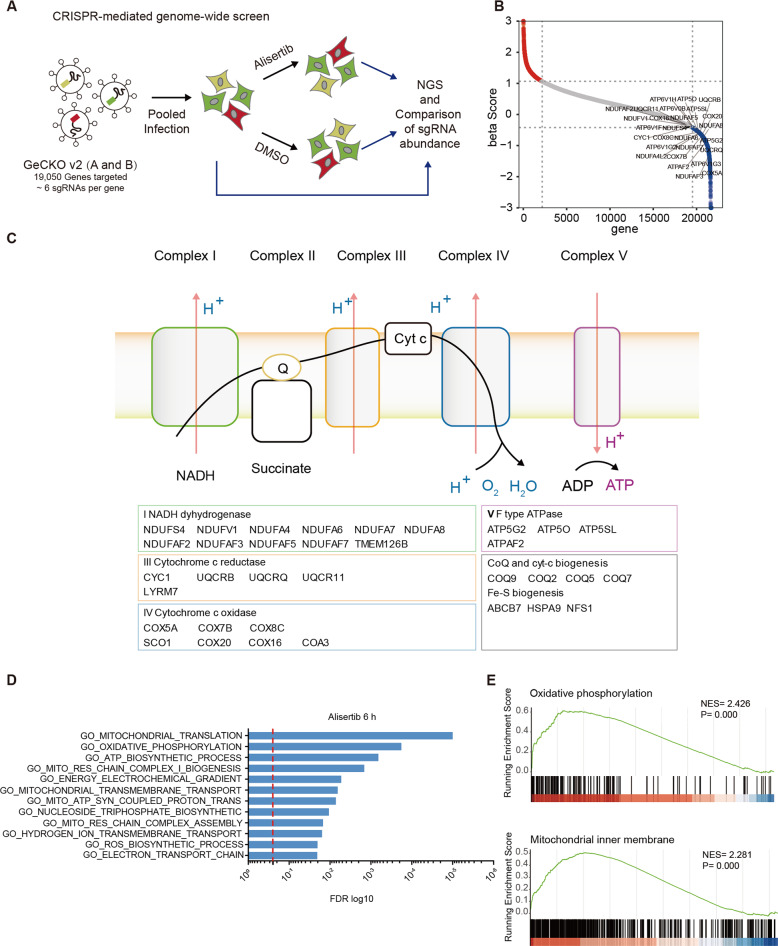
CRISPR/Cas9-based genome-wide screen reveals cellular OXPHOS-dependence upon alisertib treatment. **A** Schematic for genetic knockout screening to identify synergistic targets of alisertib in MDA-MB-231 cells. **B** Plot showing the distribution of CRISPR beta scores (treatment vs pretreatment) for genes targeted by the sgRNA library. Blue dots are the top 3000 negative enrichment genes. OXPHOS associated genes are highlighted. **C** KEGG showing significant enrichment of the OXPHOS pathway in the top 3000 negative enrichment genes. **D** GSEA analysis showing significant enrichment of OXPHOS and mitochondria respiration in the gene set that are induced upon 6 h alisertib treatment. **E** Enrichment graph of GO_Oxidative phosphorylation (NES = 2.426, *p* = 0) and GO_Mitochondrial inner membrane (NES = 2.345, *p* = 0). See also Fig. S1.

To identify the genes and biological processes that potentiate the antineoplastic effect of alisertib, we performed CRISPR/Cas9-based genome-wide screening in MDA-MB-231 cells (Fig. 1A). Briefly, MDA-MB-231 cells infected with human GeCKO sgRNA library (A and B) [11] virus were cultured with puromycin for 7 days to select sgRNA harboring cells. The sgRNA-expressing cells were then treated with the vehicle (DMSO) or alisertib. The genomic DNA of input cells and day 7 treatment/ctrl cells were extracted with no less than 4 × 10^7^ cells for each group. The sgRNA cassettes were amplified for library construction and sequencing.

The distribution histogram of median-normalized read counts in each group indicated that alisertib treatment increased the heterogeneity of the sgRNA perturbations (Fig. S1A, B). The enrichment beta scores (β scores) for each gene were calculated using the Model-based analysis of the genome-wide CRISPR/Cas9 knockout (MAGeCK) algorithm [12]. Next, we focused on the negatively selected genes of the alisertib treatment group in order to identify druggable targets that enhance the antineoplastic effects of alisertib. Surprisingly, a large set of genes that sensitize cells to alisertib are involved in OXPHOS (Fig. 1B), as the crosstalk between OXPHOS and mitosis kinase inhibitors is largely unknown. Most of the OXPHOS-associated factors that sensitized cells to the effects of alisertib are directly involved in the ETC complex I (*NDUFS4*, *NDUFV1*, *NDUFA4*, etc.), complex III (*CYC1*, *UQCRB*, *UQCRQ*, etc.), complex IV (*COX5A*, *COX7B*, *COX8A*, etc.), and complex V (*ATP5O*, *ATP5G2*, *ATP5G2*, etc.) (Fig. 1C). Factors that function in OXPHOS complex assembly and mitochondria protein synthesis are also associated with alisertib response (Fig. 1C).

To further explore the connection between OXPHOS and the alisertib response, we analyzed the transcriptome of MDA-MB-231 cells with alisertib treatment for 6 h by RNA sequencing. Gene ontology (GO) analysis of the differentially expressed gene (DEG) demonstrated enrichment in the processes of OXPHOS (FDR = 0.00018), ATP biosynthesis (FDR = 0.00066), the energy electrochemical gradient (FDR = 0.00531), and the ETC (FDR = 0.020362) (Fig. 1D). The gene set enrichment analysis (GSEA) [13] also displayed enrichment in the OXPHOS related processes including OXPHOS (NES = 2.426, *p* < 0.0001), mitochondrial inner membrane (NES = 2.281, *p* < 0.0001) as well as those associated with mitochondria respiration (Figs. 1E and S1C). In summary, the unbiased CRISPR/Cas9 whole-genome screening result and RNA sequencing transcriptome data indicates that the synthetic lethal interactions between the Aurora-A inhibitor alisertib and a specific set of genes are required for OXPHOS.

### Mitotic kinase inhibition reduces cytosolic ATP levels in mitotic cells

**Fig. 2:**
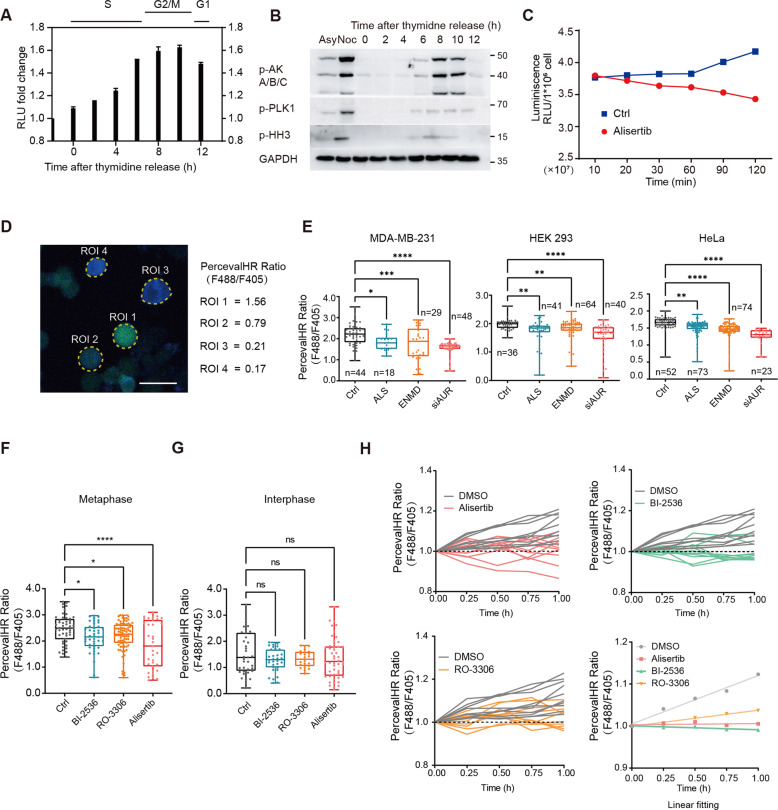
Mitotic kinase inhibition disrupts cellular energy homeostasis during mitosis. **A** ATP bioluminescent assay measuring relative ATP levels in cells released at indicated time points from a double-thymidine block. Asynchronized cells were used as controls. Mean ± SD; *n* = 3. **B** Analysis of the cell-cycle progression in MDA-MB-231 cells released from a double-thymidine block using p-HH3 (Phospho Histone H3 (S10)), p-AKA/B/C (Phospho-Aurora A (Thr288)/Aurora B (Thr232)/Aurora C (Thr198)), and p-PLK1 (Phospho-PLK1 (Thr210)). Asynchronized and nocodazole-synchronized mitotic cells were used as controls. **C** The relative ATP levels of mitotic cells were measured at the indicated time points after 100 nM alisertib treatment. Intracellular ATP levels were detected by ATP bioluminescent assay; *n* = 3. **D** Representative images of PercevalHR-expressing MDA-MB-231 cells synchronized in mitosis by nocodazole. The sensor bound to ATP (green), ADP (blue), or PercevalHR ratiometric signal is demonstrated. Scale bar, 25 µm. **E** PercevalHR ratio in mitotic cell cells after 2 h treatment with vehicle control (Ctrl), 100 nM alisertib (ALS), 5 μM ENMD-2076 (ENMD), or siRNA targeting Aurora-A (siAUR). *p < 0.05; **p < 0.01; ***p < 0.001; ****p < 0.0001. By one-way ANOVA. **F** Quantification of pH-corrected PercevalHR ratiometric signal in single cells synchronized at mitosis. All samples were treated for 2 h with vehicle control (Ctrl), 100 nM BI-2536, 10 μM RO-3306, or 100 nM alisertib in a complete growth medium before imaging. ns not significant; *p < 0.05; ***p < 0.001; ****p < 0.0001. By one-way ANOVA. Mean ± SD, (*n* = 50: Ctrl; *n* = 43: B; *n* = 80: R; *n* = 35: A). **G** Quantification of pH-corrected PercevalHR ratiometric signal in interphase cells. All samples were treated as in Fig. 2F and analyzed by one-way ANOVA. ns not significant. Mean ± SD, (*n* = 35: Ctrl; *n* = 32: B; *n* = 22: R; *n* = 48: A). **H** Live imaging of PercevalHR expressing cells synchronized at mitosis. Quantification of intracellular F488/F405 (ATP/ADP) ratios in single cells incubated in DMEM with vehicle control (DMSO), BI-2536, RO-3306, or alisertib. (Lower right) Linear fitting of F488/F405 (ATP/ADP) ratios at different time points. (The experiment was conducted on the same panel, the same DMSO group is shown in each image; The first recorded ratio was normalized to 1); *n* = 10.

Consistent with previous studies [14–16], ATP concentrations gradually increased before cell division and significantly decreased when division finished (Fig. 2A, B). The ATP accumulation process was also observed in nocodazole-treated cells (Fig. S2A). Notably, in nocodazole synchronized cells, alisertib treatment disrupted the ATP accumulation process (Fig. 2C). To scrutinize the impact of alisertib on intracellular energy homeostasis in living cells, we employed the fluorescent reporter PercevalHR [17] to monitor the ATP/ADP (fluorescence ratio of F488/F405) levels in intact cells (Fig. 2D).

To investigate whether the suppressive effect of alisertib treatment on the ATP levels is specific to Aurora-A inhibition, we treated the PercevalHR-expressing nocodazole synchronized mitotic cells with another Aurora-A inhibitor, ENMD-2076, as well as the Aurora-A targeting siRNA. Consistent with the results of alisertib treatment, a reduction in ATP/ADP ratio was observed in the cells treated with the Aurora-A inhibitor ENMD-2076 and Aurora-A targeting siRNA (Fig. 2E). This result suggests that ATP suppression by alisertib treatment is mediated through Aurora-A inhibition.

Next, we asked whether the effect of energy depletion is limited to Aurora-A inhibition. We treated the nocodazole synchronized mitotic cells with the other two mitotic kinase inhibitors, including CDK1-inhibitor RO-3306 and selective PLK1 inhibitor BI-2536. We observed a reduction of the ATP/ADP ratio in mitotic cells treated with these mitotic kinase inhibitors (Fig. 2F), however, no obvious ATP/ADP ratio change was observed after 2 h treatment of taxol in nocodazole synchronized mitotic cells (Fig. S2B). In addition, mitotic kinase inhibitors only induced reduction of the ATP/ADP ratio in mitotic cells (Figs. 2F and S2C), no obvious ATP/ADP ratio changes were observed in interphase (Fig. 2G) or Mps1 inhibitor-induced post-mitosis cells (Fig. S2D). These results indicate that the ATP reduction effect of these mitotic kinase inhibitors is limited to mitosis and is due to mitotic kinase inhibition.

To monitor the dynamics of the cellular energy level with different treatments, we performed single-cell quantification of the ATP/ADP ratio using time-lapse microscopy. Cells were synchronized at the M phase using nocodazole and reseeded in a fresh complete medium with the Vehicle (Ctrl), BI-2536, RO-3306, and alisertib independently before imaging. Analysis of the dynamic F488/F405 ratio (ATP/ADP) changes showed impaired energy accumulation with all three treatments compared with the control (Fig. 2H). And the impaired energy accumulation was restricted to mitotic kinase inhibition during mitosis (Fig. S2E, F). Our results indicate that the inhibition of mitotic kinases Aurora-A, PLK1, and CDK1 rapidly reduces the ATP/ADP level of mitotic cells.

### Mitotic kinase inhibition induces a rapid ATP loss before changes in mitochondrial respiration

**Fig. 3:**
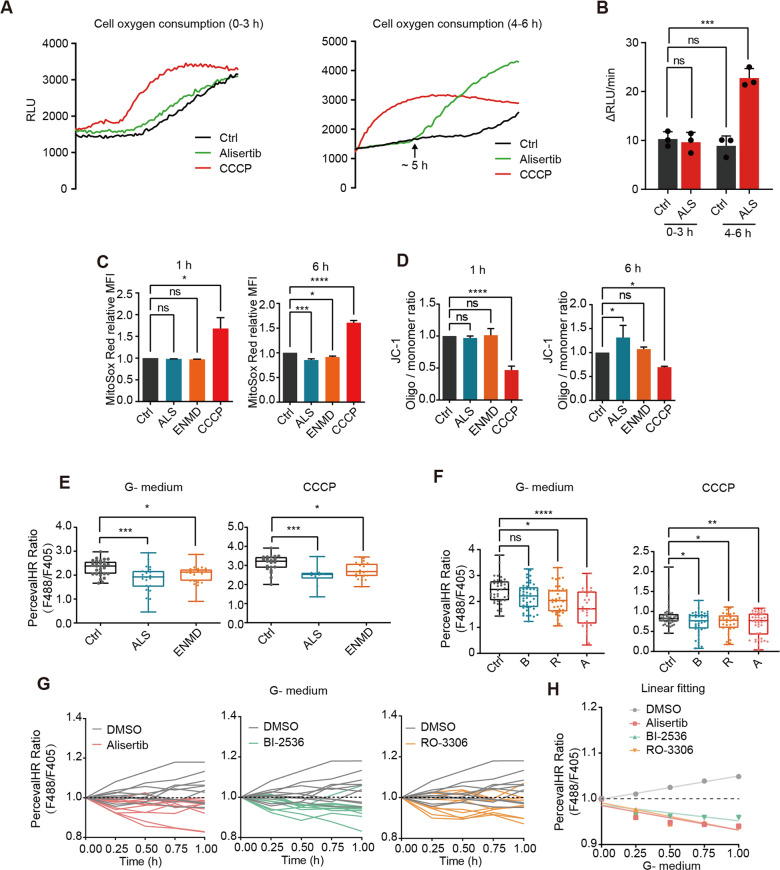
Mitotic kinase inhibition induces a rapid ATP loss before mitochondrial metabolism changes. **A** OCR curves depicting mitotic MDA-MB-231 cells treated with vehicle control (Ctrl), 100 nM alisertib (ALS), or 10 μM CCCP for 0 h (left) or 4 h (right). Relative fluorescent units (RFU) were measured in 40,000 cells per well. **B** Quantification of RFU change rates in Fig. 3A. The results are given as the mean ± SD; *n* = 3. ns not significant, ***p < 0.001 by one-way ANOVA. **C** Quantification of MitoSOX Red fluorescence intensity in nocodazole synchronized mitotic MDA-MB-231 cells by flow cytometry. Treatment for 1 h (left) or 6 h (right) with vehicle control (Ctrl), 100 nM alisertib (ALS), 5 μM ENMD-2076 (ENMD) or 10 μM CCCP. The results are given as the mean ± SD, (*n* = 2: 1 h; *n* = 3: 6 h); ns not significant; *p < 0.05; ***p < 0.001; ****p < 0.0001. By one-way ANOVA. **D** Quantification of relative mitochondrial potential via labeling with the JC-1 mitochondrial dye. Cells were treated as in Fig. 3C and analyzed by one-way ANOVA. Mean ± SD, *n* = 3. ns not significant; *p < 0.05; ***p < 0.001. **E** PercevalHR ratios in mitotic cells treated with vehicle control (Ctrl), 100 nM alisertib (ALS) or 5 μM ENMD-2076 (ENMD) in glucose-free and glutamine-free DMEM or complete growth medium supplied with 10 μM CCCP for 2 h. Mean ± SD, (left, *n* = 29: Ctrl; *n* = 22: ENMD; *n* = 26: ALS; right, *n* = 21: Ctrl; *n* = 21: ENMD; *n* = 10: ALS). Statistical analysis by one-way ANOVA. *p < 0.05; ***p < 0.001. **F** Quantification of pH-corrected PercevalHR ratiometric signals in single cells synchronized in mitosis after treatment with vehicle control (Ctrl), 100 nM BI-2536 (B), 10 μM RO-3306 (R), or 100 nM alisertib (ALS) in DMEM without glucose and glutamine or complete growth medium with 10 μM CCCP for 2 h. Mean ± SD, (left, *n* = 41: Ctrl; *n* = 44: B; *n* = 35: R; *n* = 28: A; right, *n* = 46: Ctrl; *n* = 43: B; *n* = 29: R; *n* = 42: A). The data were analyzed by one-way ANOVA. ns not significant; *p < 0.05; ***p < 0.001; ****p < 0.0001. **G** Live imaging of PercevalHR-expressing cells synchronized in mitosis. Quantification of intracellular F488/F405 (ATP/ADP) ratios in single cells incubated in glucose-free and glutamine-free DMEM with vehicle control (Ctrl), BI-2536, RO-3306, or alisertib treatment (The experiment was conducted on the same panel, the same DMSO group is shown in each image; The first recorded ratio was normalized to 1), *n* = 10. **H** Linear fitting of live-time PercevalHR ratiometric measurements in Fig. 3G.

Domenech et al. and others have reported that mitotic kinase coordinates mitochondrial dynamics with the cell cycle [18–20]. To explore whether the ATP reduction is due to synthesis inhibition or consumption acceleration, we tested the mitochondrial functions and morphological changes in the alisertib-treated mitotic cells. Surprisingly, the oxygen consumption rate (OCR) was unaffected by alisertib treatment in the first 4 h, but it doubled after 5–6 h (Fig. 3A, B). Consistent with the OCR shift, the mitochondrial membrane potential showed no detectable changes at 1 h of alisertib treatment but raised substantially after 6 h of treatment, while superoxide levels only slightly decreased after 6 h of treatment (Fig. 3C, D).

Previous studies have shown that Aurora-A and cyclin B-CDK1 regulate mitochondrial fission-fusion and ultimately mitochondrial respiration [21, 22]. Therefore, we measured the morphological dynamics of mitochondria under treatments of different mitotic kinase inhibitors. N-SIM super-resolution imaging was used to measure the quantity and size of the MitoTracker-labeled mitochondria in nocodazole-synchronized M-phase cells. None of the CDK1, PLK1, or Aurora-A inhibitors induced obvious changes in number or size mitochondria during the 1 h treatment (Fig. S3A, B, C). Thus, rapid ATP depletion by mitotic kinase inhibition may not be due to mitochondria inhibition in mitotic cells. We, therefore, considered alternative mechanisms for the reduction of ATP induced by mitotic kinase inhibition.

To understand the distinct mechanism by which mitotic kinases reduced the ATP levels in mitotic cells, cells were incubated in glucose-free and glutamine-free medium (G^−^ medium) or treated with 5 μM protonophores carbonyl cyanide m-chlorophenyl hydrazone (CCCP) to block ATP synthesis. Inhibition of mitochondrial respiration did not abolish the ATP reduction effects mediated by Aurora-A inhibition (Fig. 3E). Similar ATP reduction effects were observed in BI-2536 or RO-3306 treated cells in mitochondrial respiration inhibition conditions (Fig. 3F). Real-time quantification of the ATP/ADP ratio in G^−^ medium showed that BI-2536, RO-3306, or alisertib treatment promoted cellular ATP consumption (Fig. 3G, H). This demonstrates that Aurora-A, CDK1, and PLK1 inhibition induces rapid ATP loss before changes in mitochondrial metabolism occur.

Transcription and translation which consumes a large proportion of energy during interphase, are predominantly silenced during mitosis [23, 24]. We thus tested whether protein degradation causes ATP reduction during mitotic kinase inhibition as it is another ATP-consuming process [25]. However, alisertib treatment reduced ATP level in the presence of proteasome inhibitor MG132 (Fig. S3D–F). This suggests that the specific energy-consuming process which caused ATP reduction is yet to be defined.

### OXPHOS inhibitors potentiate the anticancer efficacy of alisertib

**Fig. 4:**
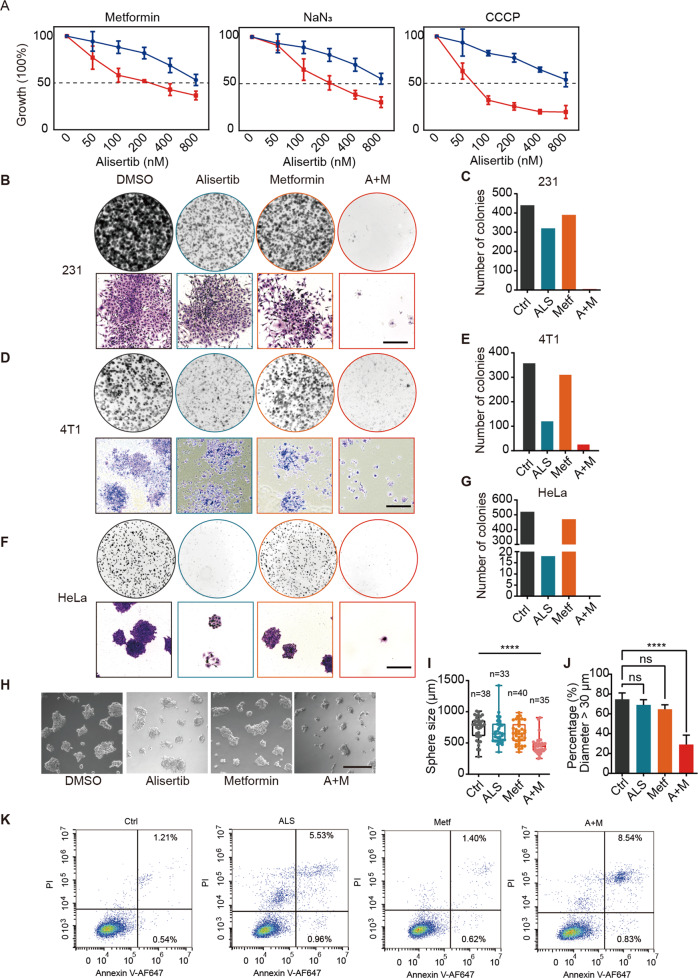
OXPHOS inhibitors potentiate the anticancer efficacy of alisertib. **A** Growth inhibition assay in MDA-MB-231 cells treated with alisertib alone (blue lines) or in combination (red lines) with mitochondrial inhibitors, including metformin (5 mM), sodium azide (1 mM), oligomycin (1 nM), or FCCP (10 mM). The relative absorbance was normalized and vehicle-treated cells were set as 100%. **B**–**G** Colony-forming assay in the MDA-MB-231 (**B**), 4T1 (**D**), HeLa (**F**) cells treated with 100 nM alisertib, 5 mM metformin, or combination (A + M). Scale bar, 500 μm. **H** MDA-MB-231 cells in 3D culture. Cells were grown in Matrigel with 100 nM alisertib, 5 mM metformin, or combination treatment. Images were taken on day 5. Scale bar, 500 µm. **I**, **J** Quantification of the diameters of cell clusters (**I**) and the percentage of cell clusters in diameter exceeded 30 μm (**J**), *n* = 3. ns not significant; ****p < 0.0001. One-way ANOVA. **K** Annexin V staining for apoptotic cells was performed in MDA-MB-231 cells after 24 h of treatment with 100 nM alisertib (ALS), 5 mM metformin (Metf), or in combination. Viable cells are in the lower left quadrant, and early apoptotic and late apoptotic cells are in the lower right and upper right quadrants, respectively.

Given that alisertib can rapidly decrease the intracellular ATP level of mitotic cells, the combination of OXPHOS inhibitors, and alisertib might further disrupt energy homeostasis in mitotic cells and enhance the antineoplastic effects of alisertib. To test this idea, we analyzed the synergistic effects of the ETC complex inhibitors metformin, Na azide, or the proton ionophore CCCP with alisertib. Metformin inhibits complex I of the mitochondrial ETC to disrupt OXPHOS and mitochondrial ATP generation [26–28]. The cytotoxic effects of alisertib were enhanced in the presence of these OXPHOS inhibitors (Figs. 4A and S4A).

Given its excellent safety profile, low cost, and minimal side effects, metformin is an attractive candidate for use alongside alisertib. The synthetic lethal interaction between metformin and alisertib was also observed in 4T1, HL60, and U937 cell lines (Fig. S4B–D). Colony formation assay showed that the combinational treatment exerted a greater suppression on the colony formation of MDA-MB-231 (Fig. 4B, C), 4T1 (Fig. 4D, E), and HeLa cells (Fig. 4F, G) compared with single agents alone. Only nondividing cells with spread-out flattened senescence-like morphologies were left with dual metformin and alisertib treatment (Fig. 4B, D, F). Single-agent administration of alisertib or metformin had minor effects on the size of the MDA-MB-231 clusters in 3D culture, whereas, the same doses in combination significantly reduced the formation of cell clusters (Fig. 4H–J). Combinational treatment of 100 nM alisertib and 5 mM metformin also resulted in greater frequencies of apoptosis as measured by annexin V/propidium iodide (PI) staining at 24 h (Fig. 4K). This shows that targeting OXPHOS via metformin administration potentiates the anticancer efficacy of alisertib.

### The combination of alisertib and metformin prolongs mitosis and increases mitotic cell death

**Fig. 5:**
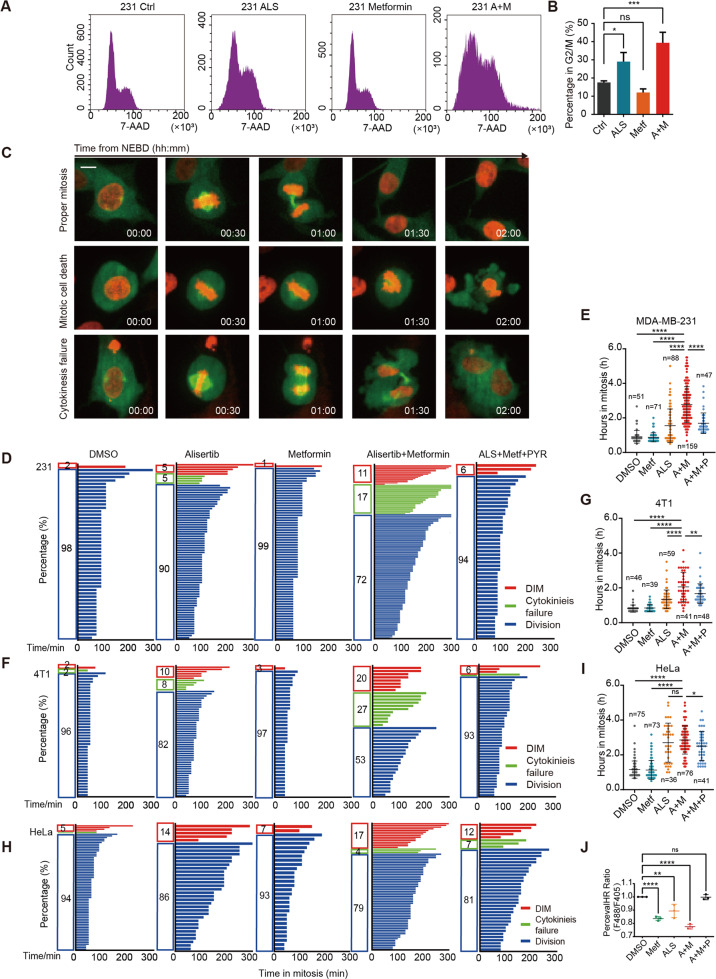
The combination of alisertib and metformin prolongs mitosis and increases mitotic cell death. **A** Cell cycle analysis of MDA-MB-231 cells treated with the indicated drugs for 24 h. **B** Quantification of 4n cells treated with the indicated drugs for 24 h in Fig. 5A. Statistical analysis was performed by one-way ANOVA. ns not significant; *p < 0.05; ***p < 0.001. **C** The representative images of cell division were recorded by time-lapse microscopy. The duration from NEBD to cytokinesis and until death or slippage was monitored. Scale bar, 5 µm. The duration from NEBD to cytokinesis or until DiM were measured and plotted as bar graph. The length indicates the duration of mitosis and its color represents the cell fate. **D**–**I** Mitosis duration and the fates of cell division recorded by time-lapse microscopy in MDA-MB-231 (**D**), 4T1 (**F**), and HeLa (**H**) cells. Cells were grown in complete culture medium with 100 nM alisertib, 5 mM metformin, combination (Alisertib + Metformin), or combination supplemented with 5 mM pyruvate (ALS + Metf + PYR). Duration of mitosis for MDA-MB-231 (**E**), 4T1 (**G**), and HeLa (**I**) cells treated with 100 nM alisertib (ALS), 5 mM metformin (Metf), combination (A + M), or combination supplemented with 5 mM pyruvate (A + M + P). **J** pH-corrected PercevalHR ratio measured by flow cytometry in MDA-MB-231 cells synchronized at mitosis, and treated with DMSO, 5 mM metformin (Metf), 100 nM alisertib, combination (A + M), or combination with 5 mM pyruvate (A + M + P). Analysis by one-way ANOVA. ns not significant; *p < 0.05; ****p < 0.0001. Mean ± SD, *n* = 3.

Metformin treatment increased the G2/M population significantly when administered together with 200 nM alisertib (Figs. 5A, B and S5A–D). To define how cancer cell responds to alisertib as a single dose and in combination with metformin, we used time-lapse microscopy to record mitosis duration and the fate of cell division. The normal division, death in mitosis (DiM), and cytokinesis failure are shown in Fig. 5C.

In the combination treatment group, the duration of mitosis was greatly prolonged from 1.5 to 2.8 h in MDA-MB-231, from 1.3 to 2.0 h in 4T1, and from 2.7 h to 2.9 h in HeLa cells, compared with the single-agent alisertib (Fig. 5D–I and Table 1). Meanwhile, the frequencies of cytokinesis failure and DiM were markedly increased in these mitotic cells (Fig. 5D–I). Cytokinesis failure resulting in binucleation and binucleation is associated with cell-cycle arrest, increased DNA damage, and cell death [29]. Our 72 h time-lapse imaging showed that the proliferation rate of binucleated cells were suppressed and the frequency of death in interphase was significantly increased by around ten times (Fig. S5E–K). In addition, DiM was slightly increased, and death in interphase take 69% of all recorded death events (Fig. S5I–K). These results elucidate that the cytotoxicity effect of cytokinesis failure is mainly caused by proliferation inhibition and cell death in the cell cycle.

**Table 1:** Duration in mitosis.

		Time in mitosis (h)					Delay (h)		Shorten (h)
		DMSO	Metf	ALS	Metf + ALS	Metf + ALS + PYR	ALS vs DMSO	ALS + Metf vs DMSO	ALS + Metf + PYR vs ALS + Metf
MDA-MB-231	Mean	0.9085	0.8756	1.545	2.771	1.691	0.6365	1.8625	1.08
	Std. deviation	0.3671	0.2623	0.9727	1.037	0.5959			
4T1	Mean	0.8116	0.8462	1.347	2.057	1.67	0.5354	1.2454	0.8116
	Std. deviation	0.2067	0.2108	0.5354	0.843	0.5665			
HeLa	Mean	1.156	1.135	2.704	2.857	2.508	1.548	1.77	0.349
	Std. deviation	0.4935	0.5441	1.121	0.8086	0.8383			

We found that a combination of alisertib and metformin further reduced ATP levels in mitotic cells (Fig. 5J). As cytokinesis failure can be induced by ATP depletion [30], we reasoned that the combination treatment causes serious energy exhaustion, thereby enhancing the killing effectiveness of alisertib during mitosis. To test this conjecture, we supplied the combination treatment cells with 5 mM pyruvate to restore the intracellular ATP levels [31]. The decrease of the cellular ATP levels was reversed by 5 mM pyruvate supplement (Fig. 5J). As expected, the mitosis duration was shortened by pyruvate supplement for 1.1 h in MDA-MB-231, 0.8 h in 4T1, and 0.3 h in HeLa cells, compared with the combination treatment of metformin and alisertib (Fig. 5E, G, I and Table 1). The above results demonstrate that the combination of metformin and alisertib further exacerbate cytokinesis failure and DiM by disrupting cellular energy homeostasis.

### Alisertib and metformin synergistically suppress breast tumor growth in vivo

**Fig. 6:**
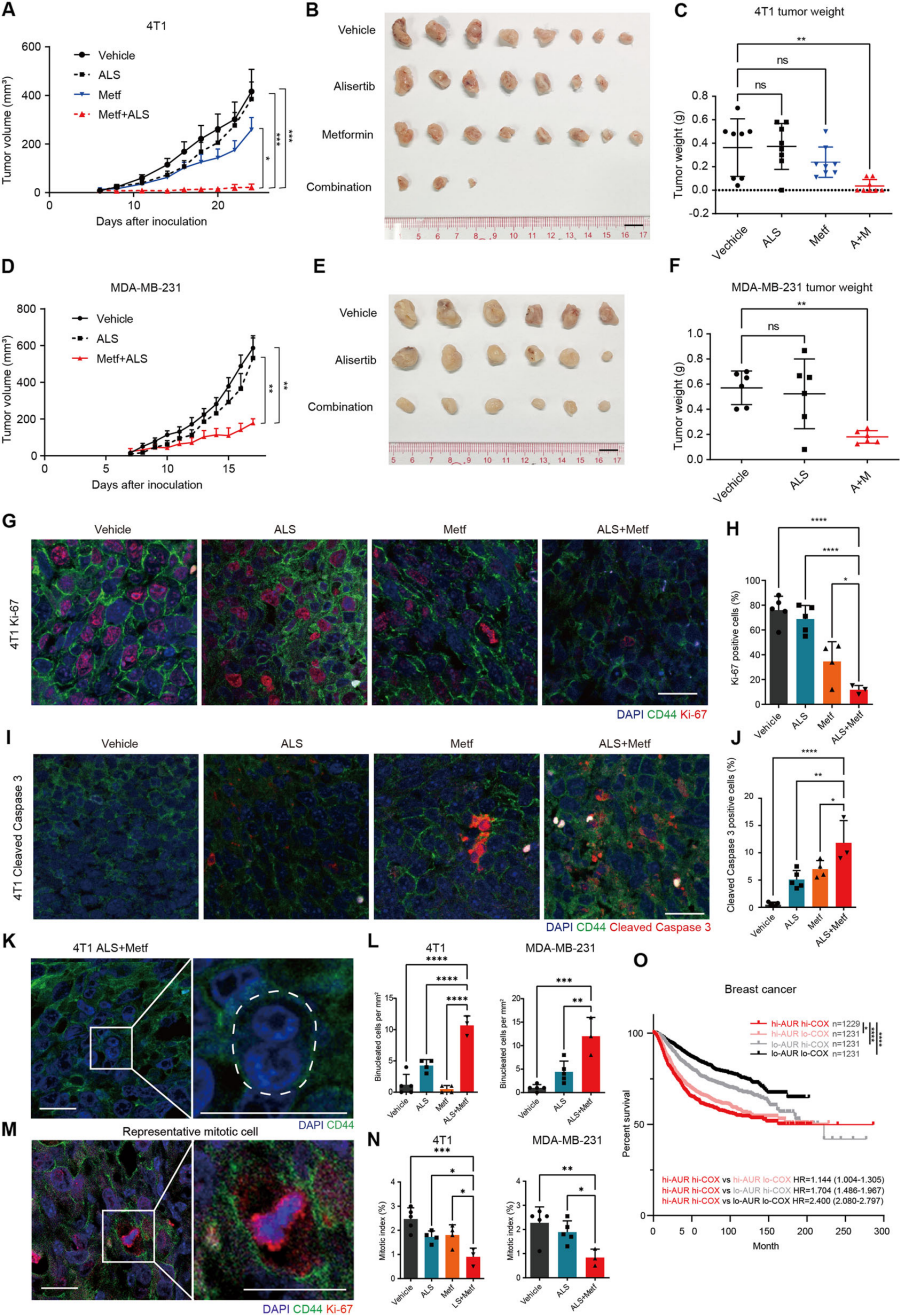
Alisertib and metformin synergistically suppress breast tumor growth in vivo. **A** The growth curves of xenograft tumors derived from 4T1 cells. Mice were subjected to daily treatments with a vehicle, alisertib (ALS), metformin (Metf), or combination (Metf + ALS). The one-way repeated-measure ANOVA followed by the least significant difference test were used to evaluate the differences between groups. *p < 0.05; ***p < 0.001. Mean + SD, *n* = 8: Vehicle, ALS, and Metf; *n* = 10: Metf + ALS. **B** Eight tumors removed from mice in each group are shown. Scale bar, 1 cm. **C** Statistical analysis of the weights of the dissected tumors. The one-way ANOVA test, followed by the least significant difference test, was used to evaluate the differences between groups. ns not significant; **p < 0.01. Mean ± SEM, *n* = 8. **D** The growth curves of xenograft tumor formed by MDA-MB-231 cells. Mice were subjected to daily treatments with vehicle, alisertib (ALS), or in combination (Metf + ALS). Statistical analysis was performed by one-way ANOVA. ****p < 0.0001. Mean + SD, *n* = 6. **E** Six tumors removed from mice in each group are shown. Scale bar, 1 cm. **F** Statistical analysis of the weights of dissected tumors. One-way ANOVA followed by multiple comparison test. ns not significant; **p < 0.01. Mean ± SEM, *n* = 6. **G** Fluorescence IHC analysis for the cell proliferation marker Ki-67 in xenograft tumors derived from 4T1 cells treated as in Fig. 6A. DNA (Blue), CD44 (Green), Ki-67 (Red). Scale bars represent 20 μm. **H** Quantification of Ki-67 positive cells in xenograft tumors samples derived from 4T1 cells treated as in Fig. 6A. The results are given as the mean ± SD (*n* = 5: Vehicle and ALS; *n* = 4: Metf; *n* = 3). *p < 0.05; ****p < 0.0001. By one-way ANOVA test, followed by the least significant difference test. **I** Fluorescence IHC analysis for the apoptotic events using anti-Cleaved Caspase 3 antibody. DNA (Blue), CD44 (Green), Cleaved Caspase 3 (Red). Scale bars represent 20 μm. **J** Quantification of Cleaved Caspase 3 positive cells in xenograft tumors samples derived from 4T1 cells treated as in Fig. 6A. The results are given as the mean ± SD (*n* = 5: Vehicle and ALS; *n* = 4: Metf; *n* = 3: ALS + Metf). *p < 0.05; **p < 0.01; ****p < 0.0001. By one-way ANOVA test, followed by the least significant difference test. **K** Fluorescence IHC analysis for binucleated cells in xenograft tumors samples derived from 4T1 cell. Images show examples from 4T1 derived tumors treated with ALS + Metf. DNA (Blue), CD44 (Green). White dashed line delineates cell border. **L** Quantification of binucleated cells in xenograft tumors samples derived from 4T1 and MDA-MB-231. For 4T1 derived tumors, the results are given as the mean ± SD (*n* = 5: Vehicle; *n* = 4: Metf and ALS; *n* = 3: ALS + Metf). For MDA-MB-231 the results are given as the mean ± SD (*n* = 5: Vehicle and ALS; *n* = 3: ALS + Metf). **p < 0.01; ***p < 0.001; ****p < 0.0001. By one-way ANOVA test, followed by the least significant difference test. **M** Representative mitotic cell revealed by fluorescence IHC analysis. Image shows an example from a 4T1-derived tumor treated with metformin. DNA (blue), CD44 (green), Ki-67 (red). Scale bar, 20 μm. **N** Quantification of mitotic cells from 4T1 and MDA-MB-231 derived tumor samples. For 4T1 derived tumors, the results are given as the mean ± SD (*n* = 5: Vehicle; *n* = 4: Metf and ALS; *n* = 3: ALS + Metf). For MDA-MB-231 the results are given as the mean ± SD (*n* = 5: Vehicle and ALS; *n* = 3: ALS + Metf). *p < 0.05; **p < 0.01; ***p < 0.001. By one-way ANOVA test, followed by the least significant difference test. **O** Relapse-free survival (RFS) for Aurora-A/COX5A transcription levels in breast cancer patients. Patient data from KM plotter is divided into four groups according to Aurora-A/COX5A expression level. HR hazard ratio. *p < 0.05; ****p < 0.0001. Log-rank (Mantel–Cox) test.

We then investigated the in vivo antitumor efficacy of metformin in combination with alisertib in xenograft mouse models. BALB/c mice bearing 4T1 xenografts were administered a vehicle, alisertib (15 mg/kg/day), metformin (300 mg/kg/day), or a combination regimen for 17 days via oral gavage. Nude mice bearing MDA-MB-231 xenografts were administered with a vehicle, alisertib (15 mg/kg/day), or the combination of alisertib plus metformin (15 mg/kg/day + 300 mg/kg/day) for 11 days by oral gavage. In agreement with the in vitro findings, the tumor volume and weight in the combination treatment group were significantly lower compared with the control or single agent administration groups (Fig. 6A–F and S6A). The body weights were slightly decreased in the combination treatment group (4% reduction in BALB/c and 6% reduction in nude mice, Fig. S6B, C).

Time-lapse imaging showed increased DiM and suppressed proliferation in a combination treatment group in vitro (Fig. 5). Hence, it is important to test whether these are also recapitulated into the tumors. We found that the proliferation marker Ki-67 was reduced in the combination treatment group (Figs. 6G, H and S6D, E). In agreement with the in vitro findings, the apoptosis marker Cleaved Caspase 3 was increased in the combination treatment group (Figs. 6I, J and S6F, G). The number of binucleated cells was also increased (Fig. 6K, L). However, unlike 24 h treatment in vitro (Fig. S5A), long time alisertib or combination treatment reduced mitotic index in tumor samples (Fig. 6M, N). The decrease of the mitotic index may be due to dropped proliferation rate in alisertib or combination treatment groups.

Lastly, we analyzed the prognostic values of *AURKA* and the OXPHOS-related gene in breast cancer using the Kaplan–Meier plotter database [32]. *COX5A*, coding a subunit of mitochondrial respiratory complex IV, is one of the top synthetic lethal genes associated with Aurora-A inhibition (Fig. 1B). As shown in Fig. 6O and S6H, I, high expression of both *COX5A* and *AURKA* significantly predicts poor relapse free survival (RFS) in breast cancer patients (HR = 2.400, log-rank *p* < 0.001, Fig. 6O).

## Discussion

**Fig. 7:**
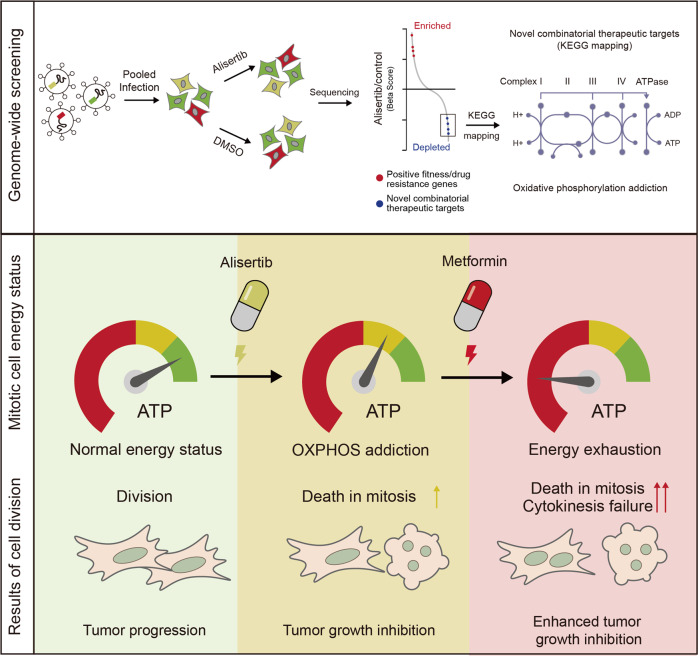
Graphic illustration of the synthetic lethal interaction between mitotic kinase inhibition and OXPHOS inhibition. Upper panel: Genome-wide knockout screening and KEGG pathway mapping of synthetic lethal targets of alisertib. Lower panel: Alisertib treatment reduces ATP concentrations in mitotic cells and OXPHOS inhibitors enhance the antineoplastic activity of alisertib through joint strike on energy homeostasis in mitotic cells. OXPHOS inhibitor treatment further reduces the intracellular ATP concentration in alisertib-treated mitotic cells, increasing the frequency of death in mitosis and cytokinesis failure.

In this study, we uncovered that mitosis kinase-targeted cancer cells display an addiction to OXPHOS due to intracellular ATP reduction using genome-wide loss-of-function screening. Consistent with this, the combination treatment of alisertib with OXPHOS inhibitors, such as metformin, increased the antineoplastic effects of alisertib in vitro and in vivo. Using a molecular ATP sensor, we validated that alisertib treatment induced a rapid ATP decrease in mitotic cells and metformin further decreased cellular energy levels in such cells. Their combination further induced cell death in mitosis and cytokinesis failure (Fig. 7). The cells are further arrested or dead in the next interphase after cytokinesis failure.

Cell division is an energy-intensive process that requires a substantial and continued supply of energy [33]. A very recent study on single-cell metabolic dependency showed that G2/M cells are more dependent on OXPHOS [34], and acute energy depletion by mitochondria inhibition leads to severe defects in dividing cells [30]. Thus, OXPHOS inhibition might synergize with the cell cycle arrest effects of mitotic kinase inhibitors, promoting further mitosis failure and DiM. As revealed by our time-lapse imaging, the combination treatment of alisertib and metformin doubled mitosis duration and increased the DiM rate compared with single-agent treatment in MDA-MB-231 cells. Replenishment of pyruvate increased the ATP level and eliminated the combinational effect of mitosis disruption in cells treated with metformin and alisertib. These results suggest that the synthetic lethal interaction between metformin and alisertib is caused by the joint strike on energy homeostasis and the subsequent increase in DiM and cytokinesis failure.

Metformin, the drug used for the prevention of type II diabetes, has proven to inhibit mitochondrial respiratory complex I [26]. It is associated with reduced cancer risk in multiple malignancies including breast, colon, pancreatic, and liver malignancies, and increased the survival rates of patients that have already developed cancer [35, 36]. However, clinical trials have shown that metformin as a single agent has either no or only marginal clinical efficacy [37, 38]. One possibility in its therapeutic potential may be part of combination therapy. Based on our findings, the combination of mitotic inhibitors such as alisertib with metformin might open novel therapeutic avenues for both stalled drugs in cancer therapy.

While we confirmed mitotic kinase inhibitors are able to induce rapid energy loss in mitotic cells, our study has not been able to pinpoint the specific biological processes that consume high levels of ATP when mitotic kinases are inhibited. It is well established that protein synthesis and related events consume half of the total energy produced in mammalian cells [39–41]. However, the transcription and protein synthesis are thought to be predominantly silenced [23, 24, 42], thus we tested whether protein degradation causes ATP reduction during mitotic kinase inhibition as it is another ATP consuming process [25]. However, alisertib treatment still reduced ATP levels in the presence of proteasome inhibitor MG132 (Fig. S3D–F). We also ruled out the effect of SAC activation by using nocodazole. Recent studies have unveiled that intracellular transport may consume a significant amount of cellular energy, especially in energy scarce conditions [43, 44]. It is possible that mitotic kinase inhibitors affect the ATP production-consumption balance; however, further investigations are required to confirm this conjecture. Based on our primary observations, mitochondria inhibition does not abrogate the effect of mitotic kinase inhibition on ATP reduction. In consequence, we propose that mitotic kinases reduce energy consumption to promote energy homeostasis during mitosis.

## Materials and methods

### Vector construction

To generate a lentiviral vector expressing ATP/ADP reporter, PercevalHR was obtained from the GW1-PercevalHR (Addgene, #49082) and inserted into the pLVX-IRES-Hyg backbone by homologous recombination. H2B and Tubulin were fused to mCherry (H2B-mCherry) and EGFP (EGFP-Tubulin) separately and cloned into the pLenti6/v5 lentiviral vector.

### Cell culture, transfection, and transduction

4T1, HEK293, HeLa, and MDA-MB-231 were cultured in Dulbecco’s modified Eagle medium (GIBCO) supplemented with 10% (v/v) fetal bovine serum (GIBCO) with 50 IU/ml penicillin and 50 mg/ml streptomycin. U937 and HL60 cells were cultured in RPMI 1640 (GIBCO) supplemented with 10% (v/v) fetal bovine serum (GIBCO) with 50 IU/ml penicillin and 50 mg/ml streptomycin and incubated at 37 °C with 5% CO_2_ in a humidified incubator. All cell lines used in this study were validated as mycoplasma-free.

Transfection was performed by using Lipofectamine 2000 (Invitrogen) according to the manufacturer’s instructions. In order to establish stable gene expression cell lines, lentivirus was produced following our previous procedure used [45]. Cells were transduced with the viral suspensions in the presence of 8 mg/ml Polybrene (Sigma-Aldrich, sc-134220) in 12-well plates. After 12 h, the lentivirus solution was replaced with fresh DMEM plus 10% FBS and seeded at 48 h into 6 cm dishes and allowed to reach confluency. Western blot was performed to measure the RNA interference efficiency [46]. Cells stably expressing PercevalHR were confirmed by confocal microscopy and purified by fluorescent cell sorting. Cells stably expressing both H2B-mCherry and EGFP-Tubulin were confirmed by confocal microscopy and selected using blasticidin S and purified by fluorescent cell sorting.

### CRISPR library screening

Lentiviral libraries were produced by transfecting Human CRISPR Knockout Pooled Library (GeCKO v2 A and B, Addgene, cat#1000000048) plasmids with pMD2.G and psPAX2 plasmids using PEI (Sigma). MDA-MB-231 cells were infected with the viral library with an MOI of 0.3 and selected with puromycin (MP Biomedicals) for 7 days. Cells were then divided into three groups: directly harvested, DMSO treated for 7 days, and alisertib treated for 7 days. For each group, the number of cells were guaranteed to be over 4 × 10^7^ to achieve 300 × coverage. Genomic DNA was extracted using an HP Tissue DNA Midi Kit (OMEGA, cat#D5197). The sgRNA cassettes were amplified to construct the libraries. The libraries were sequenced on an Illumina HiSeq2000 (150 bp, paired-end). The data was analyzed through the MAGeCK package [12].

### RNA sequencing

RNA was extracted with the HiPure Total RNA Plus Mini Kit (Magen). Library construction and RNA sequencing were constructed by Novogene with an Illumina HiSeq2000 (150 bp, paired-end). The sequencing data were qualified by fastqc (https://www.bioinformatics.babraham.ac.uk/projects/fastqc/) and the DEGs called using the RNACocktail framework [47].

### Cell growth and viability assays

For cell growth assays, 4T1, HEK293, HeLa, and MDA-MB-231 cells (1 × 10^4^ cells per well) were plated onto 96-well plates. Twelve hours after plating, the culture medium was replaced by a fresh medium with the indicated inhibitors. For U937 and HL60, 1 × 10^4^ cells were seeded into a culture medium together with the indicated chemicals. To determine cell growth and viability with different treatments, cells were studied using the Cell Counting Kit 8 assay (CCK-8). 4T1, HEK293, HeLa, and MDA-MB-231 cells were incubated in 100 μl DMEM containing 10% CCK-8 solution at 37 °C for 1 h and measured at 450 nm using a microplate reader (MD SpectraMax Plus 384). For U937 and HL60, 10 μl CCK-8 solution was added to each well, incubated at 37 °C for 2 h, and measured at 450 nm using a microplate reader (MD SpectraMax Plus 384). The absorbance reflected live cell numbers and was normalized to those in the control or vehicles and shown as relative viability (%).

### Cell synchronization

For the double thymidine block, cells were incubated with 2.5 mM thymidine for 16 h and released in a fresh DMEM culture medium for 10 h. Then, the cells were incubated with 2.5 mM thymidine for 14 h. Next, the cells were released in thymidine-free DMEM and cells or cell lysates were collected at the indicated time points. Phases of the cell cycle were analyzed by Western blotting with indicated antibodies or by flow cytometry using 7-AAD staining (see Cell Cycle Analysis).

M phase cells were synchronized using nocodazole [48] unless otherwise indicated. Briefly, cells were treated with 100 ng/ml nocodazole for 14–16 h. For western blot and ATP concentration testing, mitosis cells were collected after shake-off. For living cell imaging, mitotic cells were distinguished by cell shape and chromatin state. To induce mitotic exit, nocodazole synchronized cells were further incubated with Mps1 inhibitor MPI-0479605 for 3 h at a concentration of 1 μM. About 80 nM taxol and 5 μM MG132 treatment were used to arrest cells in mitosis.

### Cell cycle analysis

Cell cycle stages of PercevalHR expressing cells were determined by 7-AAD staining and flow cytometry. Harvested cells were adjusted to 1 × 10^6^ cells/mL and washed in cold PBS. Cells were resuspended and fixed in cold 70% ethanol at 4 °C overnight. One microliter 7-AAD of stock solution in 1 mL of cell suspension was placed on ice for 30 min for staining. PC5.5 channel of CytoFLEX Platform (Beckman Coulter) was used to detect DNA content.

The p-HH3 (Phospho Histone H3 (S10)), (Abcam, ab5176) antibody was used to determine the cell cycle state after cell synchronization and release. Cyclin B1 (Proteintech, 55004-1-AP), protein level, and aurora kinases and polo-like kinase 1 phosphorylation statue were also detected by western blot p-AKA/B/C (Phospho-Aurora A (Thr288)/Aurora B (Thr232)/Aurora C (Thr198)), (CST, 2914 S) and p-PLK1 (Phospho-PLK1 (Thr210)), (CST, 5472 T) which also indicate the specific cell cycle state. The signal was captured with Bio-Rad ChemiDoc system (Fig. 2B) and e-BLOT TOUCH IMAGER (Fig. S5C).

Hoechst 33342 (Thermofisher scientific, H3570) was used to label DNA and determine the cells in mitosis. The cells were incubated with Hoechst 33342 at 5 μg/ml concentration at 1/2000 dilution in DMEM at 37 °C. Cells with condensed chromosomes were determined as in mitosis.

### Colony formation assay

For 4T1, HEK293, HeLa, and MDA-MB-231, 500 cells were plated in each well of six-well plates and treated with candidate drugs. The medium was renewed every third day. After 1 week, cells were fixed using 4% paraformaldehyde for 15 min and stained with 1% crystal violet for 20 min at room temperature. After that, crystal violet was removed and plates were washed several times in water. The plates were photographed using ChemiDoc MP Imaging System (Bio-Rad). A representative picture was taken using a Nikon ECLIPSE Ti2 with a 10× object.

### Luminescence ATP determination

ATP concentration of mitotic cells was determined using an ATP determination kit (Beyotime, S0026), following the protocol provided by the manufacturer with minor modifications as follows. Cells were plated in six-well plates and synchronized at the indicated cell cycle state and treated by the indicated drugs before the experiment. After cell lysis, protein concentration was measured by Bradford assay, a 10 μl sample was added to 90 μl reaction solution (see product manual) in a 96-well white bottom assay plate (Corning, 3917) followed by incubation for 15 min at room temperature. Luminescence was monitored by a microplate reader (Tecan Spark, TM10M) at 560 nM at room temperature.

### PercevalHR based living cell ATP/ADP measurement

Microscope-based PercevalHR ATP/ADP level detection was performed by using laser confocal microscopy. Briefly, 2 × 10^4^ cells were seeded in a 96-well clear bottom black plate (Costar, 3603). Images were taken by a Zeiss LSM880 confocal microscope using a Plan-Apochromat 20×/0.8 N.A. M27 objective at 37 °C. PercevalHR was excited using a 488 and 405 nm laser, for the MgATP-bound conformation and ADP-bound conformation, respectively. Emission was captured through a 450–550 nm filter. pHRed was excited using a 488- and 405-nm laser, and emission was captured through a 560 nm long pass filter. Four channel image sets were taken with no delay between individual channel acquisitions. For time-lapse studies involving PercevalHR, cells were imaged on IN Cell Analyzer 2500 HS high content analysis (HCA) imaging system using a 20×/0.8 N.A. objective. Time-series images were processed using Fiji. The investigators were blinded to sample allocation.

For flow cytometry, cells were collected and resuspended in HBSS. Ratios of FITC and KO525 channels of the CytoFLEX Platform (Beckman Coulter) were used to detect ATP/ADP levels. PH-correction was performed by using a BCECF-AM pH probe (DOJINDO, B262) according to the protocol provided by the producer.

### Flow cytometry analysis of mitochondrial membrane potential and ROS production

To measure the relative levels of mitochondrial superoxide, cells were resuspended in HBSS and stained with 5 μM MitoSOX Red (Invitrogen, M36008) for 10 min at 37 °C. Cells were then washed three times with HBSS. Using a flow cytometer (CytoFLEX, Beckman), MitoSOX Red was measured using the PE channel. About 10 μM CCCP was used as a control, which increases mitochondrial ROS. Relative fluorescence intensity from biological triplicates of 10,000 cells were used as an indicator of mitochondrial superoxide levels. To measure mitochondrial membrane potential, cells were stained with a culture medium with 1× JC-1 staining solution (YEASON, 40706ES60) for 20 min at 37 °C. Cells were then washed three times with the staining buffer and subjected to flow cytometry (CytoFLEX, Beckman Coulter) following the manufacturer’s instructions. Briefly, JC-1 was excited at 488 nm and its emission at both 525 nm (FITC-A) and 585 nm (PE-A) were measured. By comparing the ratios of emission at PE/FITC, relative levels of mitochondrial membrane potential were determined from the 5000 cells in the biological triplicate. Ten micromolar CCCP was used as control which decreased mitochondrial membrane potential.

### Extracellular O_2_ consumption assay

The extracellular OCR was measured using an Extracellular O_2_ Consumption Assay kit (Abcam, ab197243) according to the manufacturer’s instructions. Briefly, 4 × 10^4^ cells were seeded in a Costar^®^ 96-well clear bottom black plate (Costar, 3603) and synchronized to M phase using nocodazole. The culture medium was replaced with fresh medium with the indicated chemicals. Ten micromolar CCCP was used as the control, which increased the OCR. Pre-warmed high sensitivity mineral oil was applied for air isolation after testing reagent was mixed with cell lysate in the wells. Signal intensity was monitored by a microplate reader (Tecan Spark TM10M) using the TR fluorescence intensity mode with the parameters: Intg1 (D1/W1), 30/100 μs, Ex 380 ± 20 nm, Em 650 ± 20 nm.

### Time-lapse imaging

A Yokogawa CV1000 Cell Voyager confocal microscope was used to record the length of time from nuclear membrane break down (NEBD) to cytokinesis and the cell fate during or after cell division. Images were collected using a 40× objective lens (40× UPLSAPO, NA = 0.95; Olympus) at seven z stacks with 7–15 μm range every 5 min. The stable incubation chamber was set at 37 °C with 5% CO_2_. We used a band-pass filter of 520/50 nm for EGFP and 617/73 nm for mCherry.

### Apoptosis analysis

Cells were treated with candidate chemicals for 24 h. The attached cells were trypsinized and collected with the supernatant. All the cells were washed with PBS twice and stained with Annexin V-AF647 Apoptosis Detection Kit (ES Science, AP006) according to the manufacturer’s instructions.

### Super-resolution live-cell imaging and mitochondrial measurements

To image mitochondria, cells were labeled with 100 nM MitoTracker Red (Thermo Fisher, M7512) for 15 min at 37 °C in phenol red-free DMEM. MitoTracker Red-labeled cells were then incubated with phenol red-free DMEM with the indicated chemicals. Super-resolution images were acquired on an N-SIM microscope (Nikon Instruments, Inc.) equipped with an Apochromat 100 × /1.49 numerical aperture oil immersion objective lens and solid-state lasers (405, 488, 561, and 647 nm). Images were captured with an ORCA-Flash 4.0 digital camera (HAMAMATSU, C11440-22CU-59) with a gain value of 100. Single plane images were processed and analyzed using Nikon Elements software using the slice reconstruction mode. The length of individual mitochondrial was traced using Fiji software, and mitochondria lengths and quantities of each mitotic cell slices were quantified. The investigators were blinded to sample allocation.

### Mouse experiments

All mouse experiments were approved by the Institutional Animal Care and Use Committee of Sun Yat-sen University Cancer Center. About 1 × 10^5^ murine mammary carcinoma 4T1 cells in 100 μl PBS were injected into the fourth mammary fat pad of 5 to 6-week-old BALB/c female mice. When the tumors reached ~3 mm in diameter, mice were randomized into four groups for treatment with alisertib only (alisertib 15 mg/kg/day), metformin only (metformin 300 mg/kg/day), a combination of metformin and alisertib (metformin 300 mg/kg/day, alisertib 15 mg/kg/day), or a vehicle (1% β-Cyclodextrin with 1% sodium bicarbonate) via oral gavage. Tumor sizes were measured every 2 days and the volumes were calculated by a previously used equation V = (length × width^2^)/2 [48].

One million luciferase-expressing MDA-MB-231 cells in 100 μl PBS were injected into the second mammary fat pad of 5 to 6-week-old nude female mice. When the tumors reached ~3 mm in diameter, mice were randomized into groups for treatment with alisertib only (alisertib 15 mg/kg/day), a combination of metformin and alisertib (metformin 300 mg/kg/day, alisertib 15 mg/kg/day), or a vehicle (1% β-Cyclodextrin with 1% sodium bicarbonate) via oral gavage. Tumor sizes were measured every 2 days and the volumes were calculated by a previously used equation V = (length × width^2^)/2 [48]. Mice were injected intraperitoneally with luciferin (3 mg per mouse, YEASEN, HB181121) and imaged using an in vivo imaging system (Bruker MI) on the 14th day after injection. The body weight of all the mice were monitored regularly before and after treatment. All mice were used in the analysis. No blinding was used.

### Fluorescent IHC

Tumors removed from mice were fixed and embedded in paraffin and sectioned for IHC. Deparaffinization/rehydration were carried out according to antibody instructions. The sections were pretreated using heat-mediated antigen retrieval with sodium citrate solution (pH6) for 10 min at 95 °C and cooled at room temperature for 30 min. The sections were blocked with 3% BSA at room temperature for 30 min and then incubated with Anti-Ki-67 (Abcam, ab15580) or anti-Cleaved Caspase 3 antibody at 1/1000 dilution at 4 °C overnight. The cells were then incubated with anti-CD44-FITC to label cell membrane at 1/300 dilution and Alexa Fluor 594 (Thermofisher Scientific, A-11037) at 1/300 dilution. Images were taken using Zeiss LSM 880 laser-scanning microscope using a 63× object operated by ZEN software.

## Supplementary information

Supplemental figure legends

Figure S1

Figure S2

Figure S3

Figure S4

Figure S5

Figure S6

Original data western blot

## Data Availability

The raw data for RNA-Seq is available in Genome Sequence Archive (Genomics, Proteomics & Bioinformatics 2017) database as accession numbers HRA001209.
