# Correction: Modulation of oxidative phosphorylation augments antineoplastic activity of mitotic aurora kinase inhibition

**DOI:** 10.1038/s41419-025-07968-4

**Published:** 2025-10-09

**Authors:** Zijian Zhang, Deshun Zeng, Wei Zhang, Ailin Chen, Jie Lei, Fang Liu, Bing Deng, Junxiao Zhuo, Bin He, Min Yan, Xinxing Lei, Shulan Wang, Eric W. -F. Lam, Quentin Liu, Zifeng Wang

**Affiliations:** 1 Sun Yat-sen University Cancer Center; State Key Laboratory of Oncology in South China; Collaborative Innovation Center for Cancer Medicine, Guangzhou, 510060 China; 2 Department of Clinical Immunology, The Third Affiliated Hospital, Sun Yat-sen University, Guangzhou, 510630 China; 3 Seventh Affiliated Hospital, Sun Yat-sen University, Shenzhen, 518107 China; 4 Institute of Cancer Stem Cell, Dalian Medical University, Dalian, 116044 China

Correction to: *Cell Death and Disease* 10.1038/s41419-021-04190-w, published online 30 September 2021

The original version of this article contains an error in Figure 6M, where the image of a representative mitotic cell was mistakenly sourced from the 4T1 Metf group rather than the 4T1 Vehicle group, as originally indicated. This image also partially overlapped with an earlier panel in the figure. The panel has now been replaced with an independent representative image from the 4T1 Metf group that is not used elsewhere. The image label has been updated to “representative mitotic cell,” and the figure legend has been revised accordingly. Original published Figure 6M legend: Typical mitotic and nonmitotic cells revealed by fluorescence IHC analysis. Images show examples from 4T1-derived tumors treated with vehicle. DNA (blue), CD44 (green), Ki-67 (red). Scale bars represent 20 μm. Revised Figure 6M legend: Representative mitotic cell revealed by fluorescence IHC analysis. Image shows an example from a 4T1-derived tumor treated with metformin. DNA (blue), CD44 (green), Ki-67 (red). Scale bar, 20 μm. These corrections do not affect any data analyses, statistical results, or the conclusions of the manuscript.

**Original published Figure 6M** 
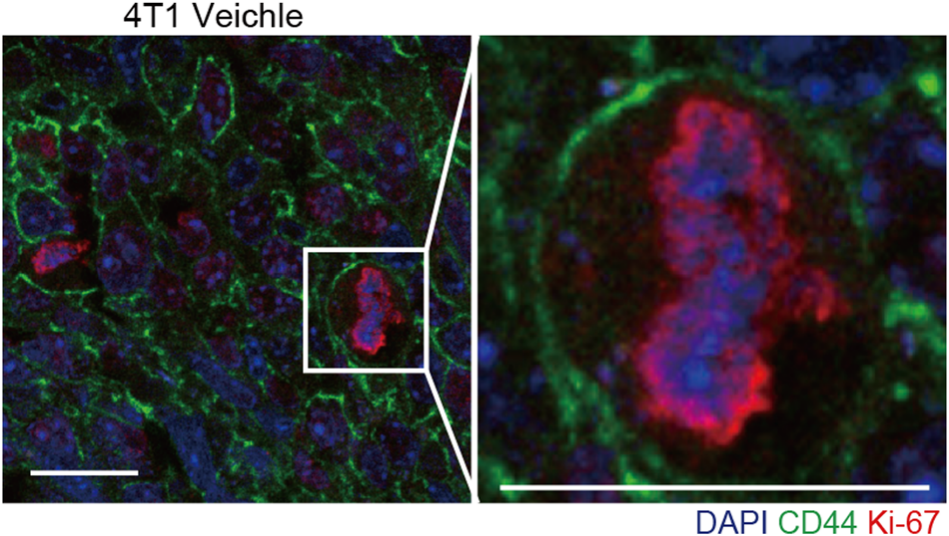


**Amended file_Corrected Figure 6M** 
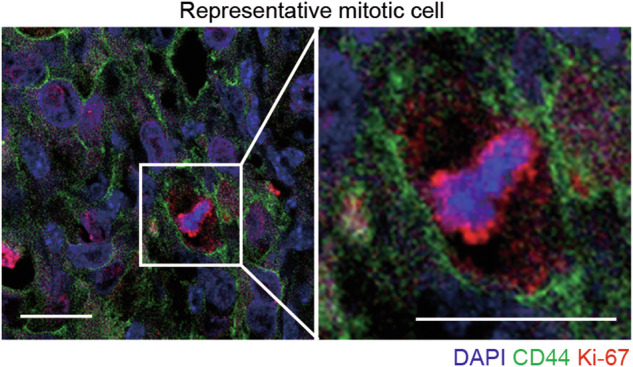


**Original data_of the Corrected Figure 6M** 
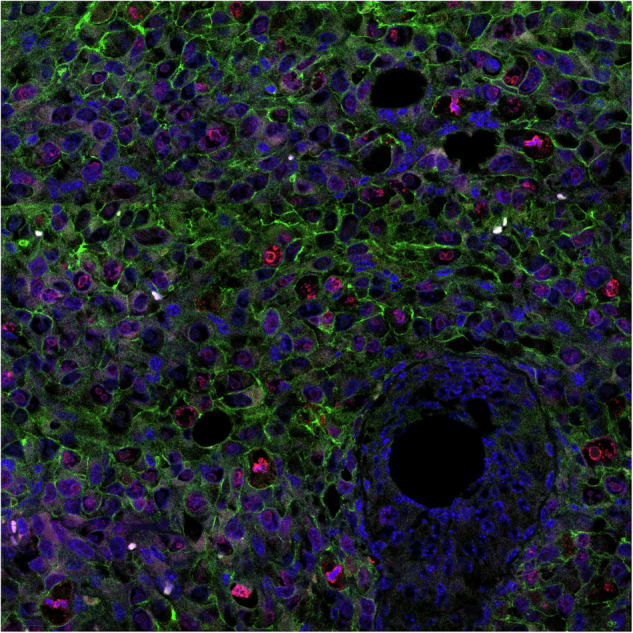


The original article has been updated.

