# Supplementary material for: Modulation of oxidative phosphorylation augments antineoplastic activity of mitotic aurora kinase inhibition
Source: Cell Death Dis. 2021 Sep 30;12(10):893. doi: 10.1038/s41419-021-04190-w (PMC8484571; doi:10.1038/s41419-021-04190-w)
Supplement: Supplementary file 1 — Supplemental figure legends [file 41419_2021_4190_MOESM1_ESM.docx]

# Supplemental figure legends

**Figure S1. CRISPR/Cas9-based genome-wide screen revealed cellular OXPHOS-dependence upon alisertib treatment, related to Figure 1.**

A. The sample clustering result.

B. The histogram of median-normalized read counts in each group.

C. Enrichment graphs of top OXPHOS and mitochondria associated gene sets.

**Figure S2. Mitotic kinase inhibition disrupts cellular energy homeostasis during mitosis, related to Figure 2.**

A. ATP bioluminescent assay measuring relative ATP levels. Cells were treated with 100 ng/ml nocodazole for the indicated times. Mean ± SD, n = 3.

B. Quantification of pH-corrected PercevalHR ratiometric signal in single cells synchronized at mitosis using nocodazole. Taxol was added 2 h before imaging at concentration of 80 nM. Mean ± SD, (n = 67: Nocodazole; n = 72: Nocodazole + Taxol). ns, not significant. Unpaired t test.

C. Quantification of pH-corrected PercevalHR ratiometric signal in single cells arrested at mitosis using taxol at concentration of 80 nM for 14 h. 100 nM Alisertib (ALS) was added 2 h before imaging. Mean ± SD, (n = 70: Taxol; n = 92: Taxol + ALS). ** *p* < 0.01. Unpaired t test.

D. pH-corrected PercevalHR ratio measured by flow cytometry in MDA-MB-231. Cells were synchronized by nocodazole and incubated with Mps1 inhibitor (1 μM, 3 h) to induce mitosis exit. Cells were then treated with control or 100 nM Alisertib (ALS) 2 h before testing. Mean ± SD, n = 3.

E. Live imaging of PercevalHR expressing cells synchronized at mitosis. Quantification of intracellular F488/F405 (ATP/ADP) ratios in single cells incubated in DMEM with control or taxol. (First recorded ratio was normalized to 1), n = 5.

F. Live imaging of PercevalHR expressing cells arrested at mitosis by taxol. Cells were treated with control or 100 nM Alisertib (ALS) before imaging, n = 5.

**Figure S3. Mitotic kinase inhibition induces rapid ATP loss before mitochondrial metabolism changes, related to Figure 3.**

A. Representation of super resolution imaging and mitochondria measurement processed by Fiji software.

B. Mitochondrial morphology visualized by N-SIM after MitoTracker red staining. The cells were treated with vehicle control (Ctrl), 100 nM BI-2536 (B), 10 μM RO3306 (R) or 100 nM Alisertib (A) for 0 and 60 min before imaging. Box indicates region shown in lower panels. The area in box were further decolorized and inverted by Fiji. Scale bars, 5 µm.

C. Quantification of mitochondria size and number in Figure S3B. The results were analyzed by one-way ANOVA. ns, not significant, * *p* < 0.05; *** *p* < 0.001; **** *p* < 0.0001, n = 10.

D. Quantification of pH-corrected PercevalHR ratiometric signal in single cells synchronized at mitosis using nocodazole. MG132 was add 2 h before imaging at concentration of 5 μM in both groups. DMSO or 100 nM Alisertib (ALS) was added at the same time. Mean ± SD, (n = 61: Taxol; n = 69: Taxol + ALS). **** *p <* 0.0001. Unpaired t test.

E. Quantification of pH-corrected PercevalHR ratiometric signal in single cells arrested at mitosis using 5 μM MG132. DMSO or 100 nM Alisertib (ALS) was added at the same time. Mean ± SD, (n = 40: MG132; n = 67: MG132 + ALS). ** *p* < 0.01. Unpaired t test.

F. Live imaging of PercevalHR expressing cells synchronized at mitosis. Quantification of intracellular F488/F405 (ATP/ADP) ratios in single cells incubated in DMEM with MG132 or MG132 plus 100 nM Alisertib (ALS), n = 5.

**Figure S4. OXPHOS inhibitors potentiates the anti-cancer efficacy of alisertib, related to Figure 4.**

A. Representative images from growth inhibition assay of MDA-MB-231 cells. Scale bar, 50 μm.

B-D. Growth inhibition assay of 4T1 (A), HL60 (B) and U937 cells (C) treated with alisertib alone (blue lines) or in combination (red lines) with metformin (5 mM for 4T1, 2 mM for HL60 and U937). Data shown are normalized to 0 mM alisertib wells in each group.

**Figure S5. Combination of alisertib and metformin prolongs mitosis and increases mitotic cell death, related to Figure 5.**

A. Mitotic and nonmitotic cells revealed by Hoechst 33342 staining. Arrowheads show mitotic cells with condensed chromosomes. Scale bar, 50 μm.

B. Quantitation of mitotic index of MDA-MB-231 cells after 24 h of treatment with 100 nM alisertib (ALS), 5 mM metformin (Metf), or in combination. Mean ± SD, n = 3.

C. Cyclin B1 protein level of MDA-MB-231 cells after 24 h of treatment with 100 nM alisertib (ALS), 5 mM metformin (Metf), or in combination analyzed by western blot.

D. Cell cycle analysis of HeLa cells treated with indicated drugs for 24 h by flow cytometry.

E, F. Time-lapse imaging showing cell division frequency of MDA-MB-231 cells expressing ER-GFP and H2B-mcheery treated with 100 nM alisertib and 5 mM metformin. Arrowhead indicates binucleated cell (Binuc). Histogram showing division events recorded in 20 h per 100 cells. Mean ± SD, n = 3. ** *p* < 0.01. Unpaired t test.

G, H. MDA-MB-231 cells dead in next interphase treated with 100 nM alisertib and 5 mM metformin. Arrowhead indicates binucleated cell (Binuc). Histogram showing probability of death in interphase. Mean ± SD, n = 3. ** *p* < 0.01. Unpaired t test.

I, J. MDA-MB-231 cells treated with 100 nM alisertib and 5 mM metformin dead in next mitosis. Arrowhead indicates binucleated cell (Binuc). Histogram showing probability of death in mitosis. Mean ± SD, n = 3. * *p* < 0.05. Unpaired t test.

K. Pie chart showing proportions of death in next mitosis and death in next interphase of binucleate cells recorded by 72 h time-lapse imaging.

**Figure S6. Alisertib and metformin synergistically suppress breast tumor growth *in vivo*, related to Figure 6.**

A. Representative bioluminescence images show tumor burden formed by MDA-B-231 on the 14th day after inoculation.

B, C. Body weight of BALB/c mice (B) and Nude mice (C) on the day of sacrifice. * *p* < 0.05, by one-way ANOVA.

D. Fluorescence IHC analysis for the cell proliferation marker Ki-67 in xenograft tumors derived from MDA-MB-231 cells treated as in Figure 6D. DNA (Blue), CD44 (Green), Ki-67 (Red). Scale bars represent 20 μm.

E. Quantification of Ki-67 positive cells in xenograft tumors samples derived from MDA-MB-231 cells in Figure S6D. The results are given as the mean ± SD (n = 5: Vehicle and ALS; n = 3: ALS + Metf). * *p* < 0.05; **** *p* < 0.0001. By one-way ANOVA test, followed by the least significant difference test.

F. Fluorescence IHC analysis for the apoptotic events using anti-Cleaved Caspase 3 antibody. DNA (Blue), CD44 (Green), Cleaved Caspase 3 (Red). Scale bars represent 20 μm.

G. Quantification of Cleaved Caspase 3 positive cells in xenograft tumors samples derived from MDA-MB-231 cells in Figure S6D. The results are given as the mean ± SD, n = 5. * *p* < 0.05; **** *p* < 0.0001. By one-way ANOVA test, followed by the least significant difference test.

H, I. Relapse-Free Survival (RFS) for breast cancer patients stratified by Aurora-A or expression level COX5A alone using the Kaplan–Meier plotter online tool. HR, hazard ratio.
