# Supplementary figures and images for: Modulation of oxidative phosphorylation augments antineoplastic activity of mitotic aurora kinase inhibition
Source: Cell Death Dis. 2021 Sep 30;12(10):893. doi: 10.1038/s41419-021-04190-w (PMC8484571; doi:10.1038/s41419-021-04190-w)

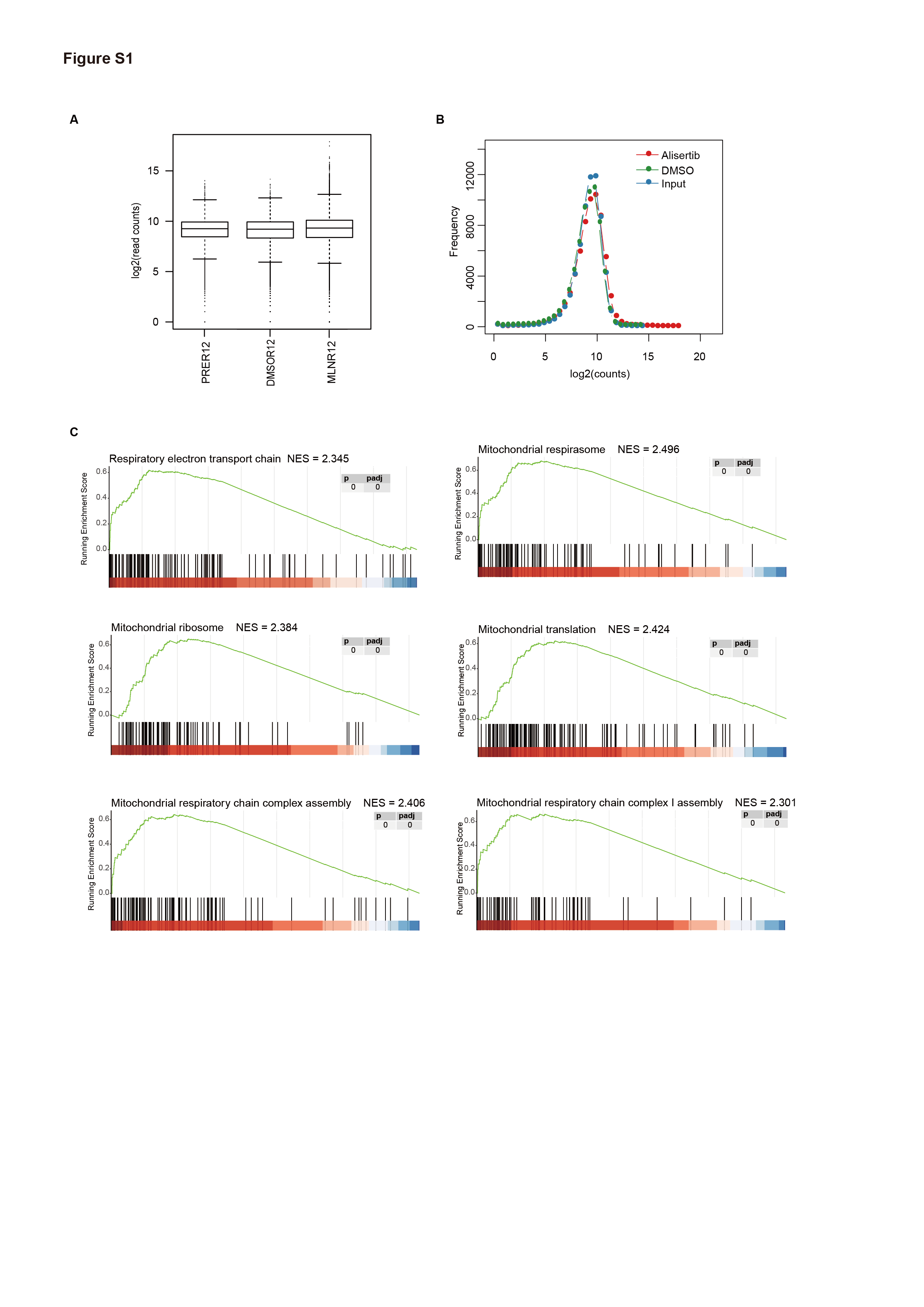

Supplement: Supplementary file 2 — Figure S1 [file 41419_2021_4190_MOESM2_ESM.png]

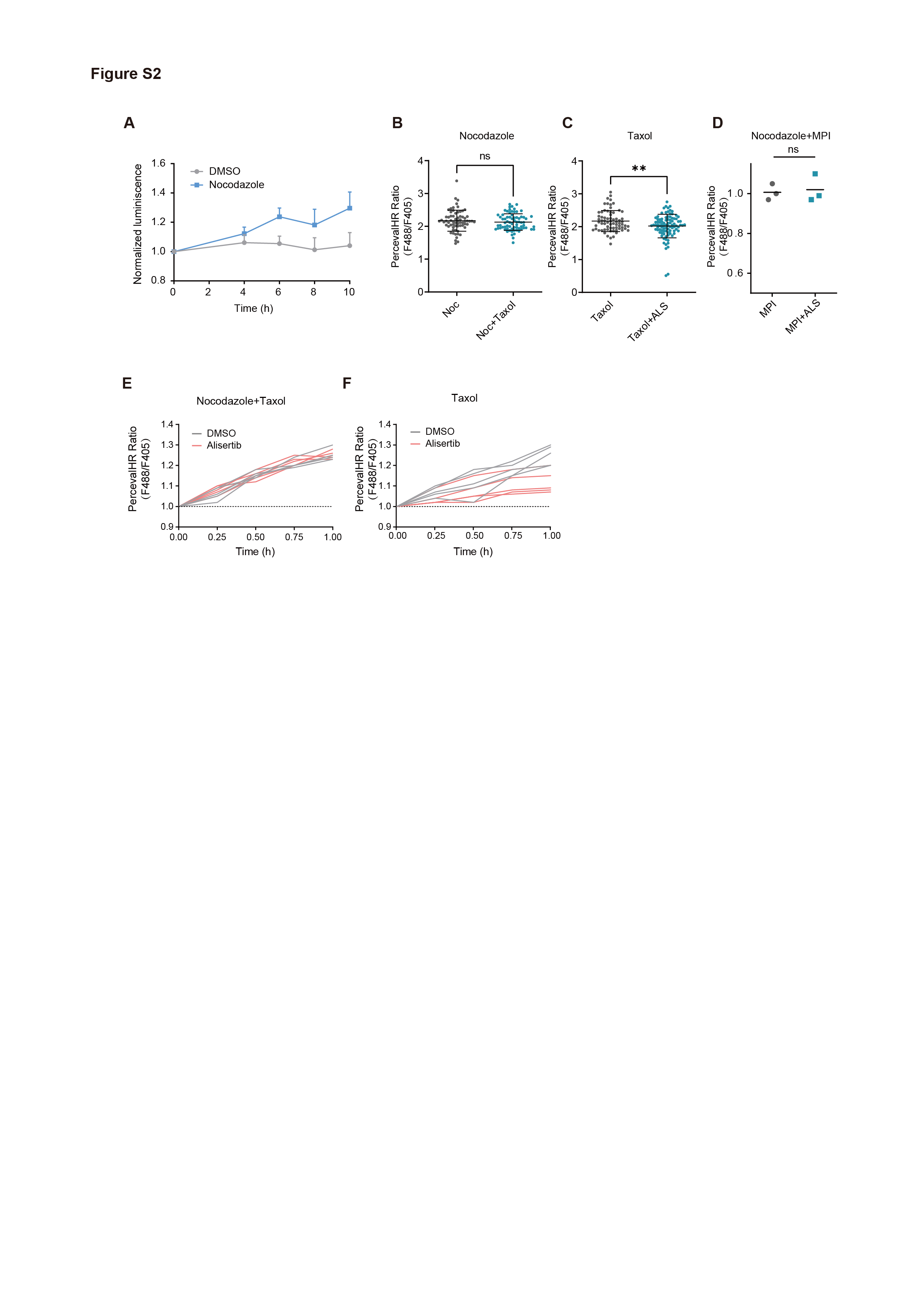

Supplement: Supplementary file 3 — Figure S2 [file 41419_2021_4190_MOESM3_ESM.png]

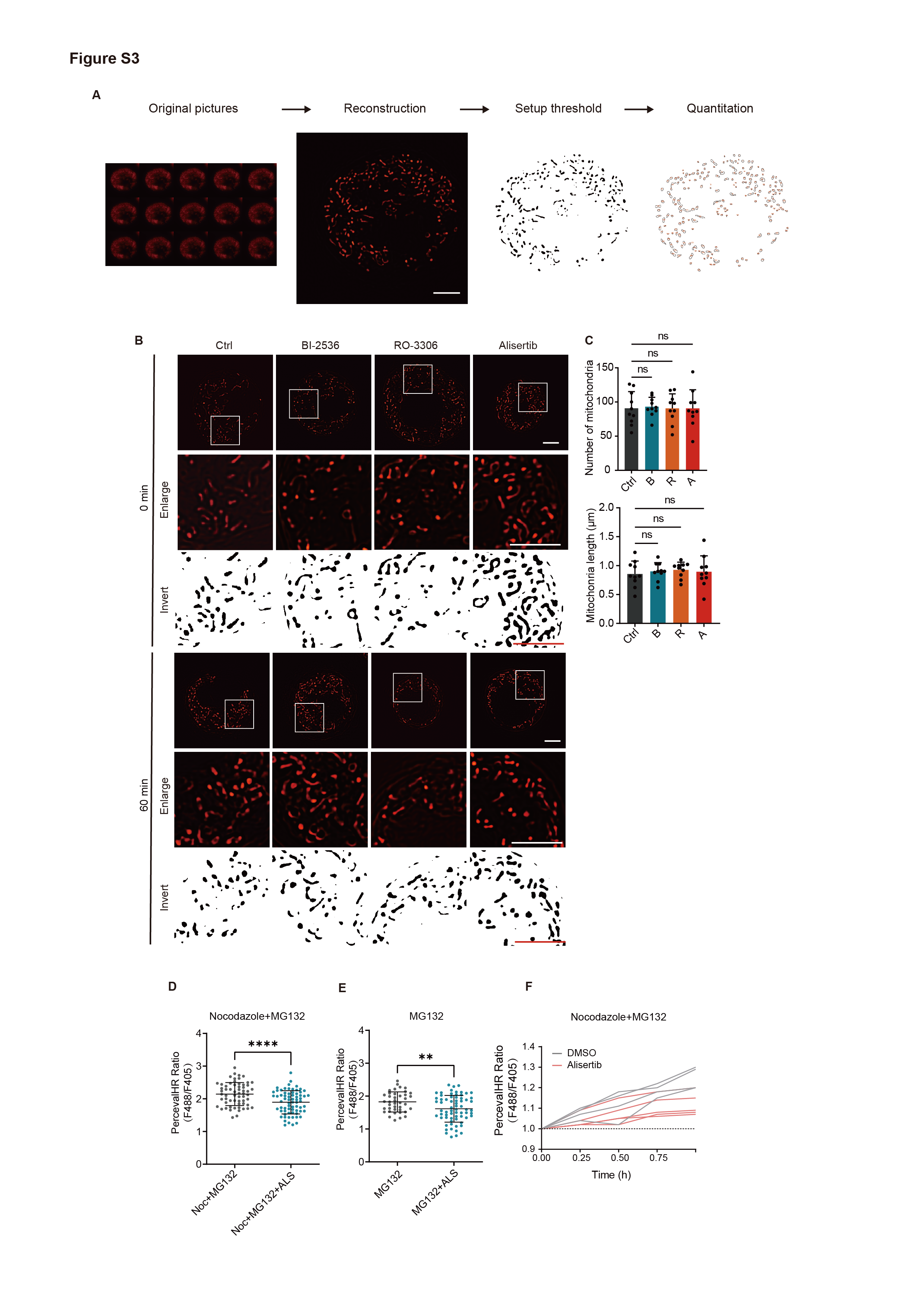

Supplement: Supplementary file 4 — Figure S3 [file 41419_2021_4190_MOESM4_ESM.png]

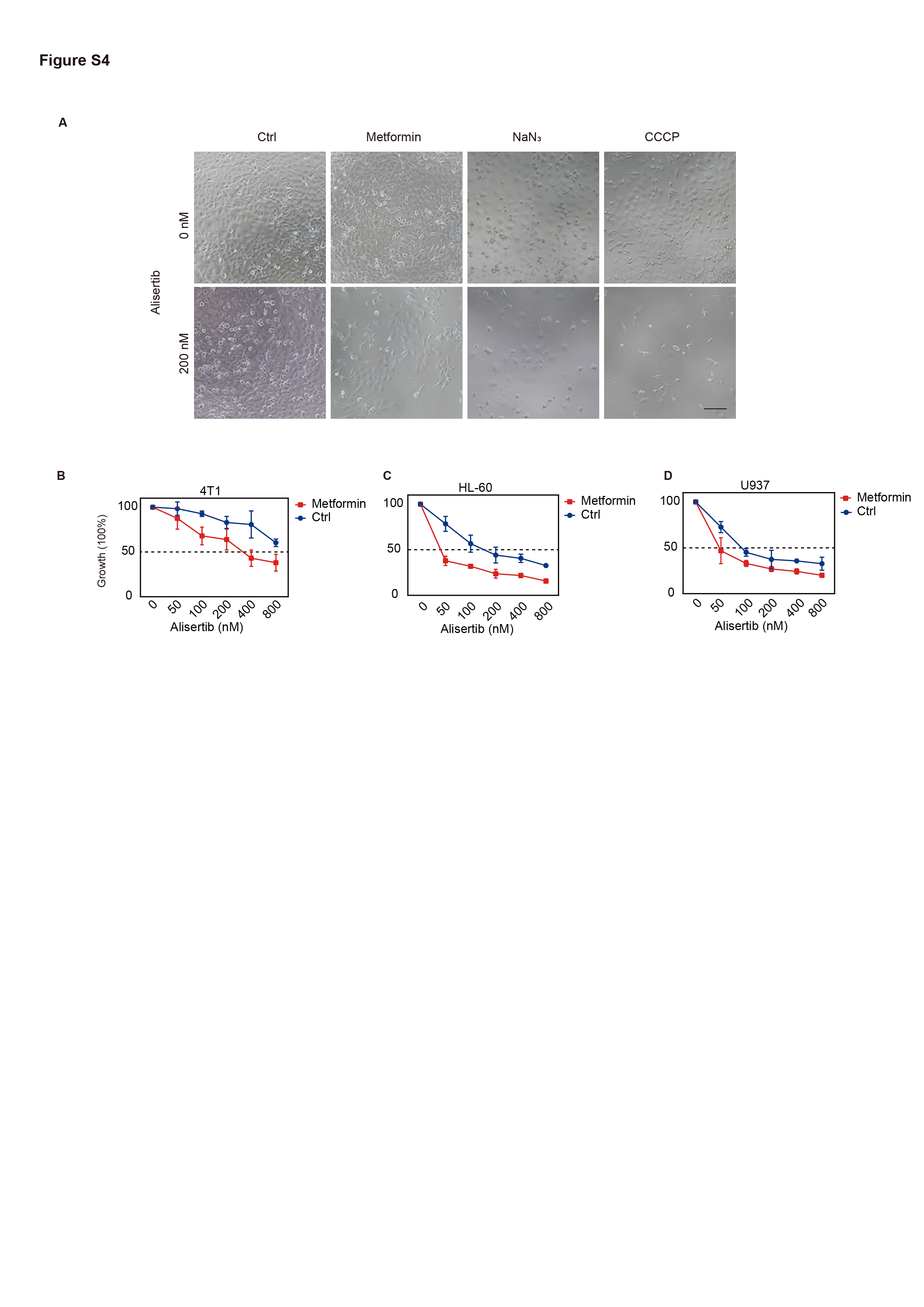

Supplement: Supplementary file 5 — Figure S4 [file 41419_2021_4190_MOESM5_ESM.png]

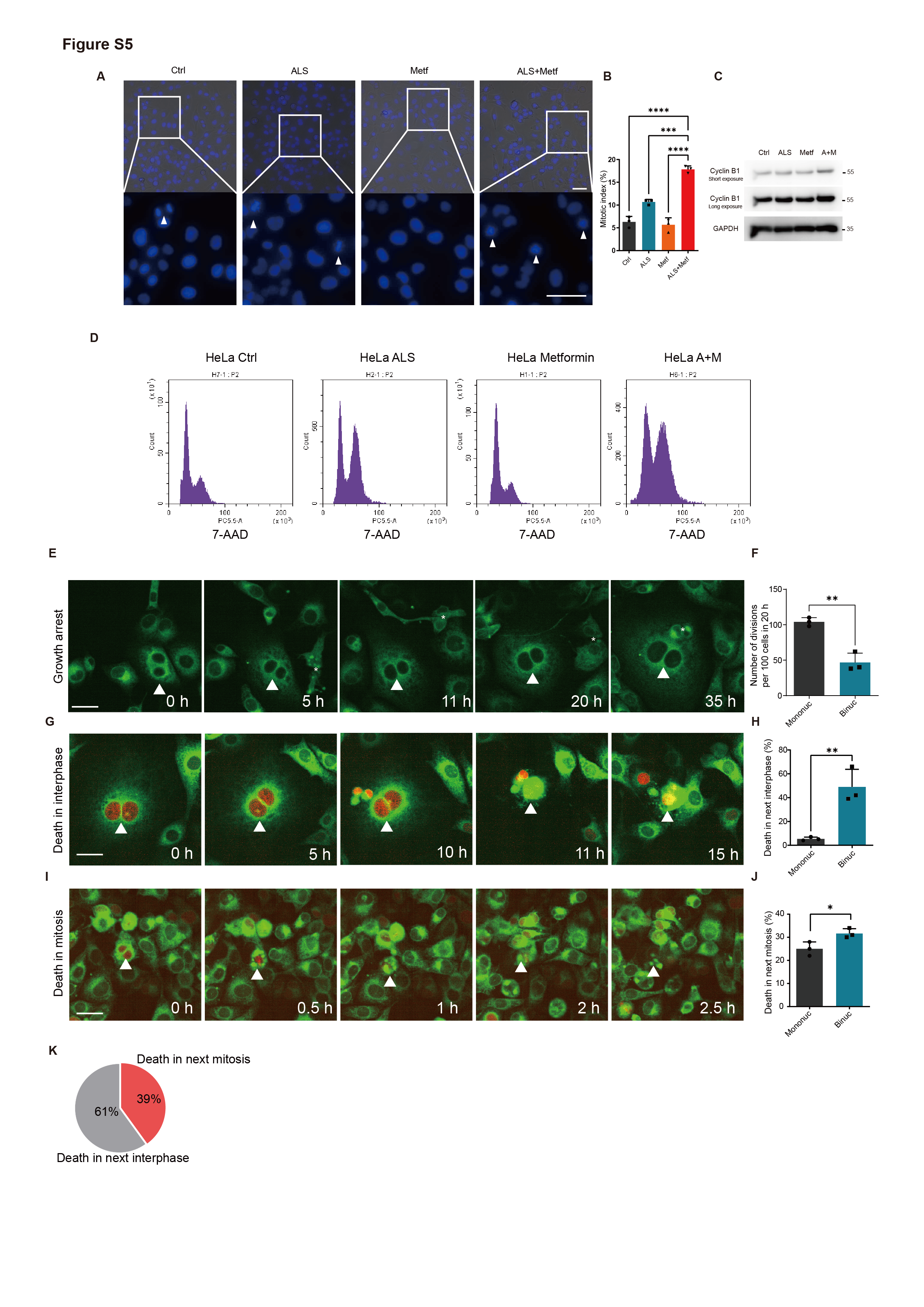

Supplement: Supplementary file 6 — Figure S5 [file 41419_2021_4190_MOESM6_ESM.png]

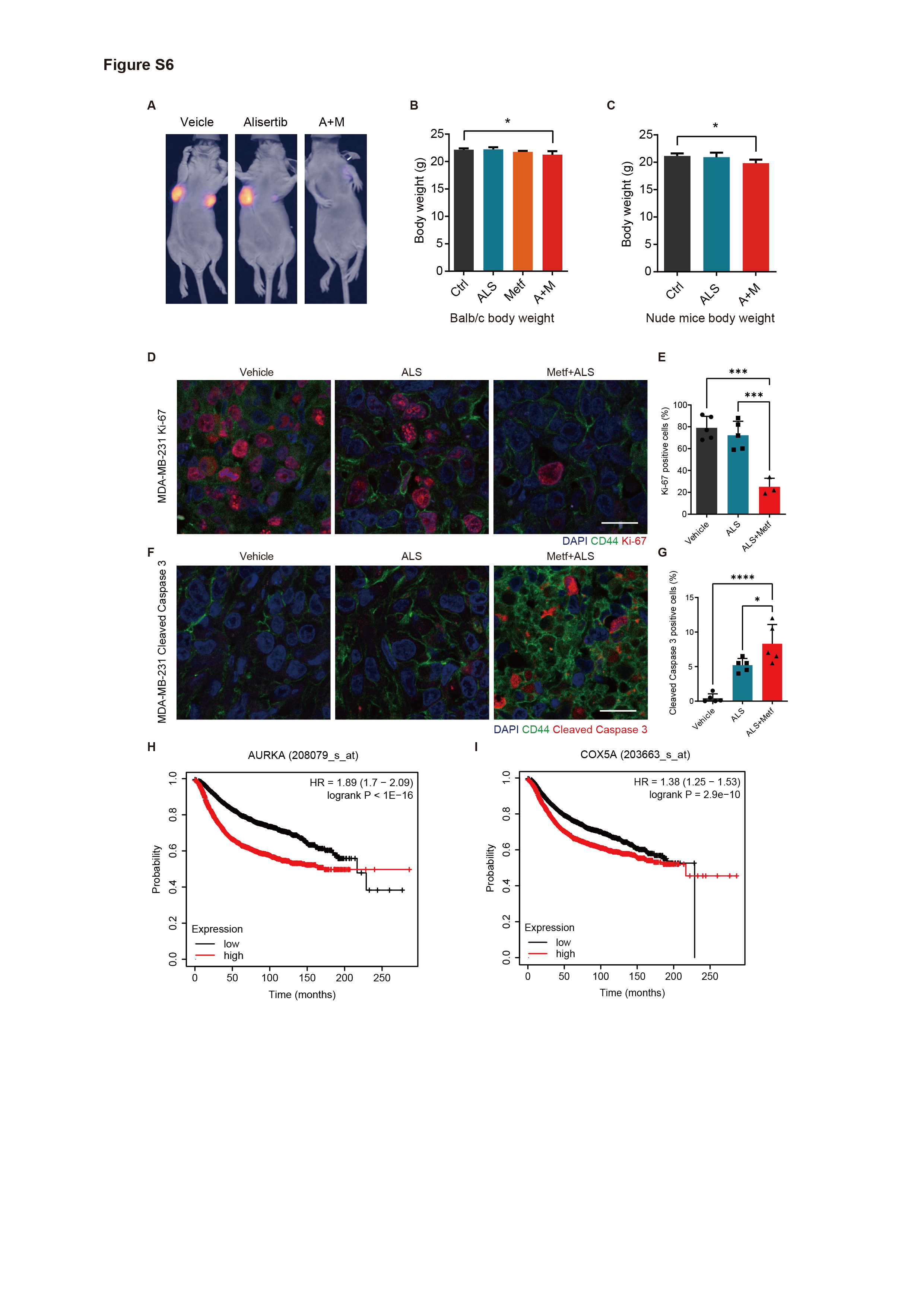

Supplement: Supplementary file 7 — Figure S6 [file 41419_2021_4190_MOESM7_ESM.png]

Figure 2B

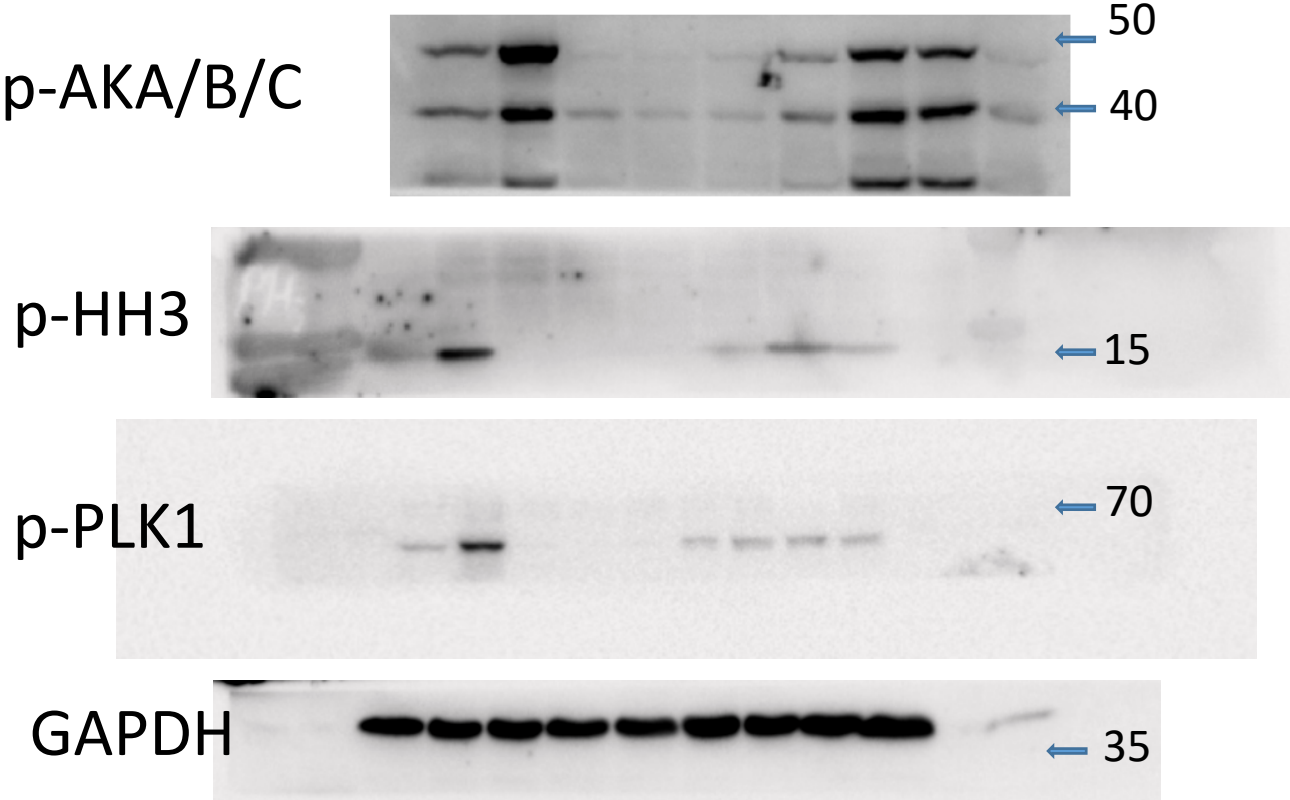

Figure S5C

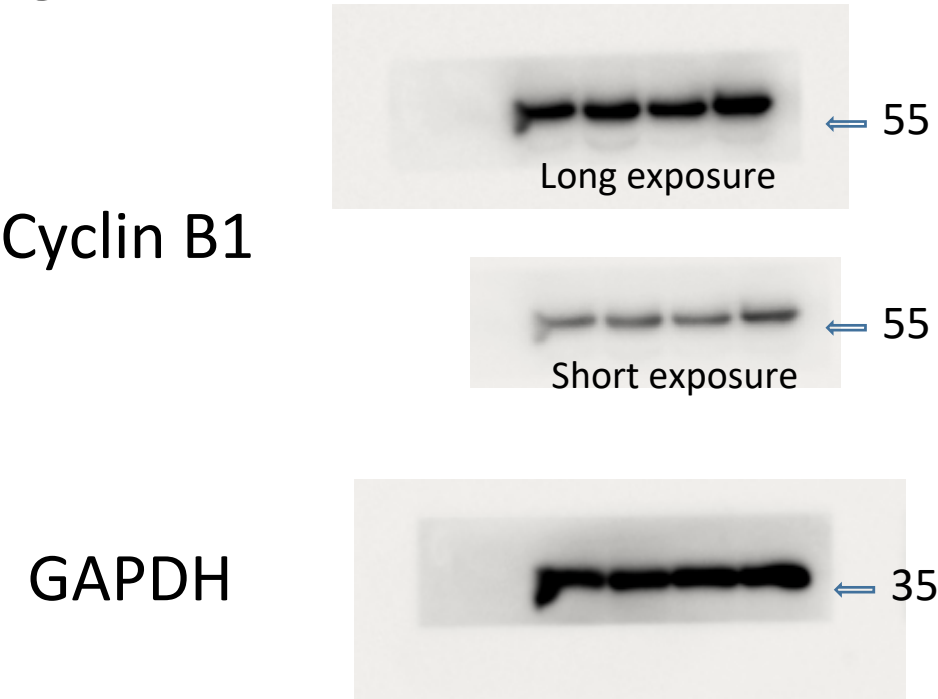

Supplement: Supplementary file 8 — Original data western blot [file 41419_2021_4190_MOESM8_ESM.pdf]
